# High-resolution lithostratigraphy and reconnaissance sedimentology of Changotaung structure, Chittagong Tripura fold belt, Bengal Basin, Bangladesh

**DOI:** 10.1038/s41598-023-43810-7

**Published:** 2023-10-18

**Authors:** Noshin Sharmili, Saiful Islam Apu, Md. Yousuf Gazi, Md. Anwar Hossain Bhuiyan, Janifar Hakim Lupin

**Affiliations:** 1 Department of Geology, University of Dhaka, Dhaka, 1000 Bangladesh; 2 Department of Geosciences, The Pennsylvania State University, University Park, PA 16802 USA; 3 Department of Geology, The University of Kansas, Lawrence, KS 66045 USA; 4 School of Geosciences, Faculty of Science, University of Sydney, Sydney, Australia

**Keywords:** Geology, Petrology, Sedimentology

## Abstract

Unlike other structures in the vicinity of the Chittagong Tripura Fold Belt, the Changotaung anticline is one of Bangladesh's least explored structures. An attempt has been made for the first time to understand and document sedimentary deposits, environments, structure, and tectonic activity based on the high-resolution outcrop and reconnaissance study with the knowledge of broad-brush geology. We found that Changotaung is a symmetrical box-folded structure with an extensive western flank where the amount of dip varies between 11° and 45°. The exposed Cenozoic succession was categorized into three separate sedimentary sequences and correlated with the conventional stratigraphic unit. A first-order simple Markovian approach was presented for the exposed litho-section in an effort to illustrate vertical facies variations in the Upper Surma group. We quantified that heterolithic bed mostly overlies both trough cross-bedding ($${P}_{ij}U$$ = 0.706) and parallel laminated bed ($${P}_{ij}U=0.955)$$ according to the facies transition probability matrix. According to the results of the stationary distribution, there is a 40% chance of coming across heterolithic beds within the Upper Surma group during any given event that is completely random whereas trough cross-bedding, parallel laminated bed, cumulative sandstone facies, and cumulative shale facies shows around 10.8%, 15.2%, 20.6%, 13.4% probability. We hypothesized, based on the interpretive facies analysis, that the Chittagong Tripura fold belt region's Upper Surma Group underwent three interrelated depositional settings (wave-dominated shallow marine, tide-dominated shallow marine, and fluvio-deltaic distributary).

## Introduction

Chittagong Tripura Fold Belt (CTFB) is the farther-west continuation of the Indo-Burma Range (IBR) and resides in close proximity to the convergence of the Indian and Burmese plates making this a highly earthquake-prone zone^1,2^. The common folded structures observed within the eastern compressed fold thrust zone of CTFB include Barakal, Belasari, Gobamura, Kasalang, Shishuk, Utan Chatra, Sakudaung, and Banuachari. Conversely, the western fold thrust area comprised Sitakund, Sitapahar, Bandarban, Changotaung, Semutang, Dakhin Nhila, Inani, Matamuhari, Lambaghona, Olathang, and Jaldi structures^3^. Scholars investigated different structures of the CTFB region to decipher natural hazard and hydrocarbon assessments. Comprehensive sedimentological investigations of the outcrop-based logging, sequence stratigraphy, provenance analysis, and two-dimensional structural modeling of the Sitakund anticline were executed to understand the depositional scenario of Miocene sediments, hydrocarbon system, and associated configuration in SE Bangladesh^4,5^. Seismic studies alongside the lithostratigraphic and geophysical well-log analysis indicated that the elongated Semutang anticline revealed a convexly curved flank in the east and a relatively steep flank in the west^3,6^. Besides, the anticline has an analogous fold axis to the Halda structure, and upper gas sand (UGS) has been apprehended in the Middle Miocene to Late Miocene formation^6^. Facies analysis, facies models, and sedimentary and geomorphic features have been broadly studied along the Sitapahar anticline to evaluate the depositional environment of the Surma group^7–9^. This asymmetric open-folded structure exhibits a double plunge, and Neogene succession concurred progradation sequences from deep marine to fluvial deposits followed by shallow marine events^9,10^. Descriptive outcrop sediments of the Bandarban structure were studied to evaluate the impact of tectonics, climate, and transgressional and regressional events on the depositional settings^11,12^. Jaldi, Dakhin Nhila, and Inani structures have moderate amplitude, and seismic studies indicated the possibility of restraining from the fold initiation^13^. Major portions of Inani and Dakhin Nhila anticlines have been obscured by the Chittagong Coastal Fault (CCF) that accommodates the stress related to the convergence of the Indo-Burma plates^3,14,15^.

**Figure 1:**
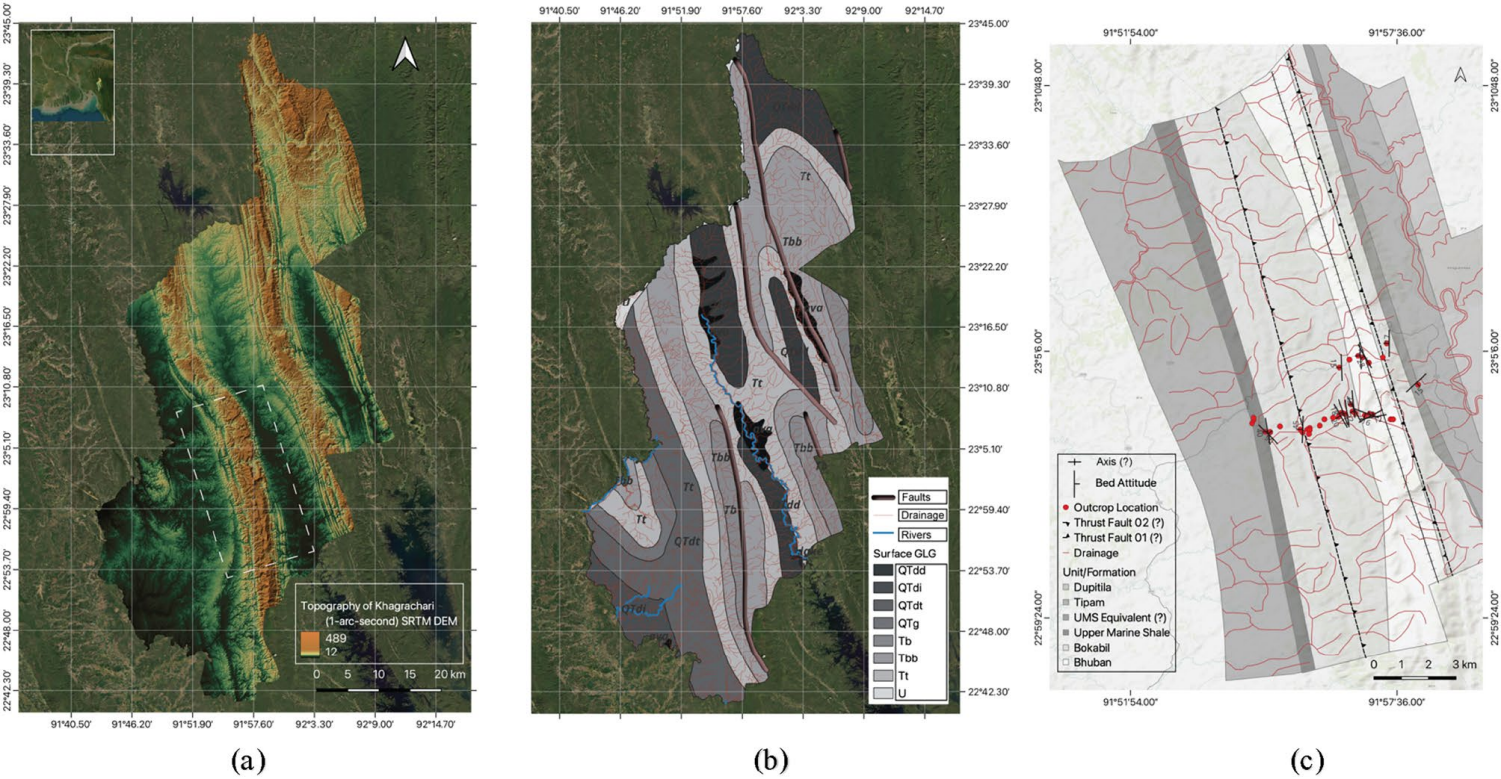
(**a**) Location of the Changotaung structure (Dashed white box between Matiranga and Khagrachari Town) at Khagrachari. (**b**) Surface geological expression in Khagrachari within the CTFB region^16^. (QTdd = Undivided Dihing and Dupi Tila Fm.; QTdi = Dihing Fm. ; QTdt = Dupi Tila Fm. ; QTg = Girujan Clay; Tt = Tipam Sandstone; Tbb = Bokabil Fm. ; Tb = Bhuban Fm.). (**c**) The generalized focused geological map of Changotaung Anticline where Alutila-Risang-Thakurchara and Risang-Dhoilachora-Bangmarasetu sections were explored to determine the region's geology explicitly. The question mark (?) emphasized that a more detailed revised investigation is required to comprehend the features accurately. A schematic cross-section has been provided in the supplementary information. All the map has been generated using QGIS, version 3.30 (https://www.qgis.org/en/site/) with the SRTM-downloader plugin (https://plugins.qgis.org/plugins/SRTM-Downloader/).

Albeit the structures in the western fold-thrust zone (WFTZ) have been studied from various aspects, the Changotaung anticline got less attention due to its remote location and inaccessible locality (Fig. 1). Most of the anticlines of WFTZ are in the Chittagong and Bandarban districts. In contrast, Changotaung is situated within the Khagrachari district, one of the least explored areas compared to the other regions of CTFB. One study attempted to decipher the fold axis's orientation and the Changotaung structures' shortening alongside the other edges of the IBR^3^. To infer lithofacies analyses, stratigraphic successions, and the depositional environment from the exposed rocks, thorough first-hand research has been conducted on the Changotaung anticline structure. The conclusive contrast between the compressed eastern and western folded thrust zone within CTFB has yet to be distinct conclusively. The present study is the first detailed geological investigation in Khagrachari (part of CTFB) which can serve as a baseline study for the Changotaung structure in the context of facies analysis. We tried to provide a Markovian analysis of the exposed litho-section which is also the first known attempt to date for the Surma Group yet it is one of the single most important stratigraphic succession of the Bengal Basin. This evidently helped us to decipher a new possible depositional scheme for the Upper Surma Group and the constraints of conventional depositional settings in Chittagong Tripura Fold Belt and Sylhet Trough. The present study attempts to present comprehensive geological information on this area to create more scopes for a further comprehensive inspection for regional and local interest. Previously, adjacent structures, i.e., Sitakund, Sitapahar, and Semutang, have proven their higher potentiality of hydrocarbon. Moreover, the Chittagong and Rangamati region got more attention for the landslide susceptible study although Khagrachari also resides in the same geologically active tectonic zone. Hence, it is crucial to understand the sedimentary system, stratigraphic records, lithology, and depositional environment to shed light on this region's hydrocarbon potentiality and risk management. Moreover, the provided Markovian method in this study can be widely used to depict vertical facies variation in any regional geological locality.

## Regional geology and tectonic setting

The Bengal Basin is postulated to have undergone evolutionary processes on a foundation of remnant ocean basin and rifted continental frontier of eastern India, with NW parts being underlain by continental crust and its S and SE parts being underlain by oceanic lithosphere^17,18^. Bangladesh's stable platform in the NW and geosynclinal basin in the SE are separated by an elongated NE-SW paleo-continental slope^10,19,20^. The stable platform is split between the Bogra shelf, which has a substantial sedimentary deposit (~ 1–6 km), and Rangpur Saddle, which has a thin, limited sedimentary cover (~ 130–1000 m) towards the north^21,22^. In addition to this, the geosynclinal basin can be split into two parts: the Fold Belt to the east and the Foredeep to the west. The eastern fold belt has more vigorous tectonic activity, as evidenced by the succession of anticlinal (upward folds), synclinal (downward folds), and thrust faults. However, the magnitude of the fold exhibition lessens as one travels towards the west, where the fold belt unit fuses with the foredeep entity, which is characterized by only minimal folding or no folding at all^20,23,24^.

The establishment of stratigraphic units in Bangladesh was carried out utilizing the lithostratigraphic approach, with particular emphasis on the Tertiary strata that have been exposed within the Lower Assam Basin^25^. Multiple investigations were performed to partly develop this earlier scheme based on micropaleontology, palynology, lithofacies, and seismic stratigraphy^26,27^. The current understanding of the regional contrast between the Bhuban and Bokabil formations within the Surma Group lacks sufficient definition, thereby emphasizing the necessity for an updated stratigraphic framework.

The Bengal Basin's geological evolution since the Gondwanaland breakup in the late Mesozoic, spanning across Bangladesh and the eastern region of India is still active^28^. The Chittagong-Tripura Fold Belt (CTFB) has revealed a substantial accumulation of clastic sedimentary rocks belonging to the Surma and Tipam Group. The inferred depositional settings for the Surma Group succession have been widely regarded as a paradigmatic representation of the stratigraphic, tectonic, and sedimentological history of the Bengal Basin^10,29,30^. CTFB exhibits a close association with the eastward subduction of the Indian plate within the framework of an arc-trench tectonic configuration and is considered the outer portion of the Indo-Burman Range^31,32^. During the temporal span encompassing the Late Cretaceous up until the conclusion of the Oligocene, it is observed that the Indian plate persistently engaged in the process of subduction beneath the west Burma block. This particular geological phenomenon gave rise to the formation of the entirety of the Indo-Burma Range (IBR), which serves as a testament to the progressive augmentation and translocation of an accretionary prism^33^. The prism complex was moving farther west through the CTFB region (coined as Neogene accretionary prism) and developed the Indian lithosphere^34^. Structures in the CTFB are presumably governed by the accretionary wedge and involve considerable high-angle thrusting. A certain amount of disharmonic folding can be seen in these structures: Firstly, an inconsistency in the breadth of synclines and anticlines; secondly, box-like and ridge-like morphology in the anticlines; and thirdly, substantial amplitude and echelon couplings^31,34^. It should be emphasized that because the trench axis is getting farther away from the original line of subduction, the ophiolitic blocks found in the IBR would be less prevalent (or occur at a lower stratigraphic horizon) in the CTFB^35^.

## Methods

For understanding and presenting a comprehensive geological framework of folded Neogene succession of South-eastern Bangladesh, detailed reconnaissance fieldwork has been carried out on the Changotaung structure. The study comprises highly calibrated outcrop-based sedimentary logging for facies and facies association, standardized numerical modeling, and simulation.

Data and measurements were obtained from the exposed sections along the road cut, inside the cave in Matiranga Upazila, and nearby Khagrachari City using systematic litho-stratigraphic descriptions and columns^36^. For lithofacies assessment (especially for facies associations), sedimentary structures, textures, unit/formation thickness, and possible contiguity have been snapped, documented, and depicted in graphical logs^5,37–39^. Different sub-section logs (vertical and lateral) were correlated according to their representative facies and facies association, allowing the identification of other stratigraphical units and depositional environments. The graphical representations of the litho-columns and geological map were followed according to USGS symbolization and cartography guidelines by the Federal Geographic Data Committee (FGDC)^40^.

A generalized Markov chain analysis has been performed to get statistical insights into the vertical facies sequences^41–43^. This mathematical technique (Markov Model) is a representation of the stochastic process that yields sequences of events with a given frequency^43,44^. Although there are multiple analytical techniques proposed for the Markov model, in this study, a first-order modified Embedded Markov Chain (EMC) has been employed, including facies transition probability matrices, stationary distribution analysis, transitional diagram, Markov model chain computation, and simulation^45–49^. This EMC technique has been modified and performed in accordance with the following equations^41,50,51^.

Transition probability matrix: ${P}_{ij}U={F}_{ij}/{R}_{i}$

Here, $${F}_{ij}$$ is the facies transition count and $${R}_{i}$$ is the corresponding sum of the row. We determined the upward transition probability matrix ($${P}_{ij}U)$$ here. For the downward transition probability matrix $${(P}_{ij}D)$$ it can be computed by dividing $${C}_{i}$$ which is the corresponding sum of the column.

Stationary distribution: $$\psi {P}_{ij}U=\psi$$

Here, the Markov chain's stationary distribution has been performed to reconcile unconstrained and single-dimensional processes alongside the single-step facies transition. Eigenvalue and eigenvector have been computed and normalized from the transition probability matrix.

Markov model chain computation and simulation: $P\left\{{X}_{t+1}\right\}=\mathrm{min}\left\{k|c\left({X}_{t},k\right)\right\}\ge {r}_{ij}$

Here, computation is fast and effective while it takes transition probability, initial state, and sequence length as the input rather than entirely computing random probability matrix, deducing different matrix, and expected frequency matrix. The modeling simulation referred to the next time step based on the defined initial state. The cumulative sum of the transition probability matrix has also been defined such that $c\left({X}_{t},k\right)= \sum_{ij=0}^{t=1}{P}_{ij}U$.

## Results and interpretation

### Lithofacies analysis and specification

**Figure 2:**
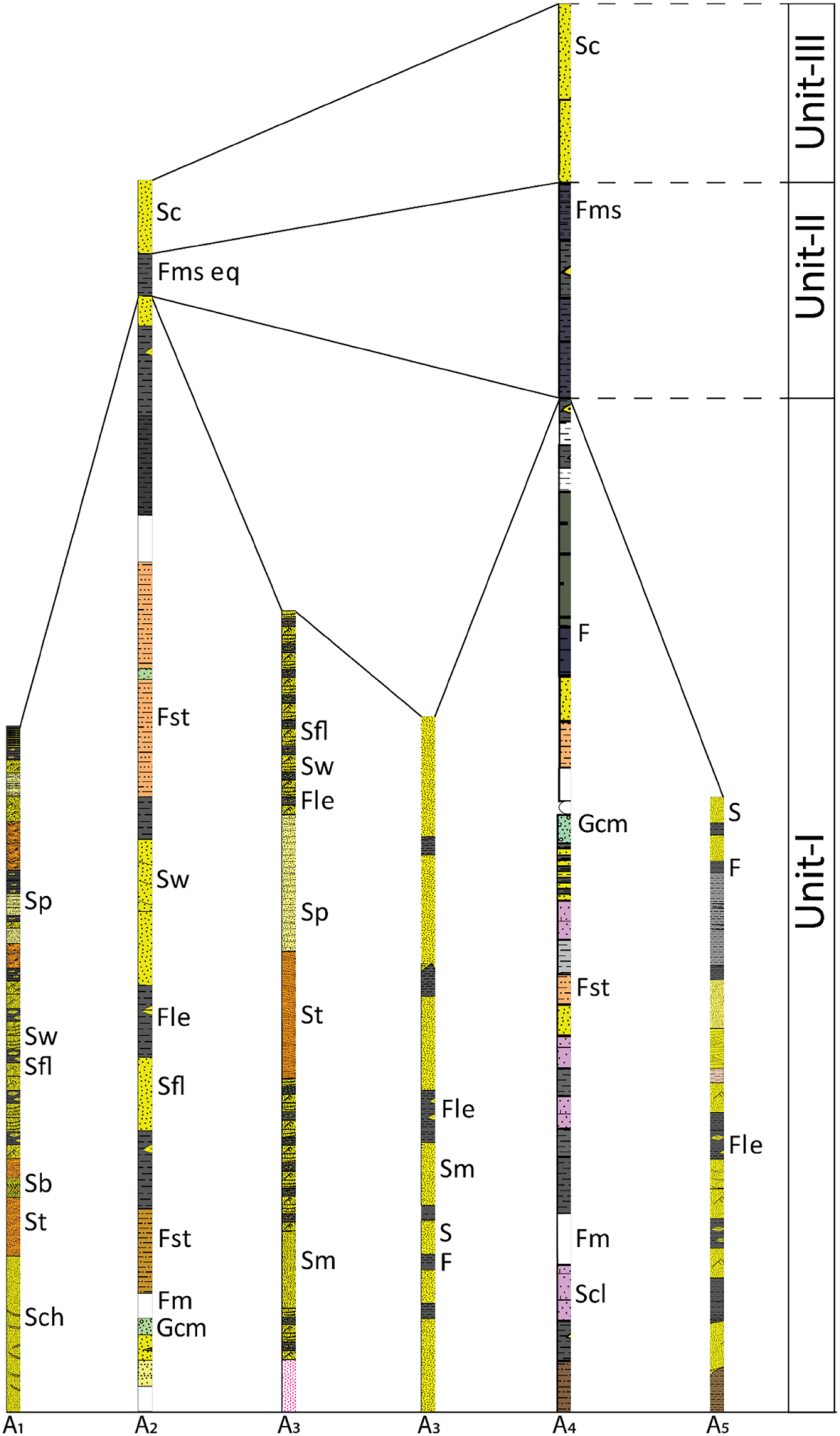
Summarized lithofacies of exposed rock sequence of Changotaung structure. Based on the litho correlation, the units were depicted synthetically according to the interpreted facies association. Corresponding unit/Formation (I): Surma Group from Miocene in age, Corresponding unit/Formation (II): Upper Marine Shale from Mio-Pliocene in age, Corresponding unit/Formation (III): Tipam Sandstone from Pliocene in age.

Geological aspects were investigated through the Alutila-Risang-Thakurchara section and the Risang-Dhoilachora-Bangmarasetu section. We assemblage five facies associations (Table 1) and decode them into different facies primarily based on the sedimentary features, structures, grain size, and lithology (Fig. 2).

**Table 1:** Identified lithofacies from the outcrop sections at Risang waterfall, Risang-Thakurchara, Alutila cave and Army camp, Dhoilachara-Bangmara, and Matiranga-Alutila Road Cut sections.

Facies association	Facies code	Facies types (cumulative)
A_1_	A_1_a	Herringbone, hummocky, trough cross-bedding followed by heterolithic bed
	A_1_b	Parallel laminated bed, cross-bedded sandstone, heterolithic bed, and sand-shale alteration
A_2_	A_2_a	Claystone, parallel laminated bed, and sandstone with clay lenses
	A_2_b	Ex-situ Conglomerate
	A_2_c	Claystone, lenticular bed followed by sandstone with clay streak
	A_2_d	Repetitive feature of flaser, lenticular, and wavy bedding
	A_2_e	Shale with silt followed by minor pieces of conglomerate
	A_2f_	Claystone and lenticular bed with occasional exfoliated shale
	A_2_g	Flaser, wavy, lenticular bed with a yellowish sandy streak
A_3_	A_3_a	Flaser, lenticular, wavy bedding followed by massive sandstone, trough cross-bedding, parallel laminated bed
	A_3_b	Sand injected into the clay with overlain load cast followed by occasional lenticular bed
A_4_	A_4_a	Claystone, lenticular, and calcareous band with occasional shale
	A_4_b	Siltstone, nodular shale, calcareous band underlain over a massive sandstone
	A_4_c	Repetitive sand-shale alteration with in-situ para-conglomerate and concretions
	A_4_d	Claystone and siltstone overlay on a massive sandstone
	A_4_e	Fissile shale
	A_4f._	Paper-thin laminated shale
	A_4_g	Pro-deltaic shale with frequent lenticular bed
	A_4_h	Marine shale
	A_4_i	Yellowish brown massive sandstone
A_5_	A_5_a	Graded bedding followed by flaser and lenticular
	A_5_b	Heterolithic bed with clay
	A_5_c	Shale with a silty streak
	A_5_d	Trough cross-bedded with parallel laminated bed and massive sandstone

### Markov model

The Markov model of this study has been confined to the Risang waterfall and Alutila cave section where the well-exposed lithology can be used to construct depicted vertical facies variation in the Surma group. The combination of three lithologs allowed us to see any significant facies transition in the succession. Table 2 gives us the one-step observed facies transition count from the exposure where the row and column represent the current states and next states of the facies respectively. A total of 141 transitions have been counted in the system which includes St, Htb, Sp, S, and F. The probability matrix shows that both St (~ 0.7) and Sp (~ 0.95) are mostly overlain by Htb which is again highly overlain by Sp (~ 0.3). This represents a continuous transition between high and low energy conditions, and it can be more accurately seen between S(Sch/Sb/Sm) and F(Fm/Fst) where each other overlain by ${P}_{ij}U$ value.

**Table 2:** Lithofacies transition of Markov matrices in the exposed section of the Risang and Alutila section which have been deciphered as the Surma group.

Lithofacies	St	Htb (Sfl/Fle/Sw)	Sp	S (Sch/Sb/Sm)	F(Fm/Fst)
i)	Facies transitional count matrix ($${F}_{ij})$$				
St	0	12	3	2	0
Htb (Sfl/Fle/Sw)	3	0	4	4	2
Sp	0	42	0	1	1
S (Sch/Sb/Sm)	2	24	0	0	13
F(Fm/Fst)	1	11	2	14	0
ii)	Facies transitional probability matrix ($${P}_{ij}U)$$				
St	0.000	0.706	0.176	0.118	0.000
Htb (Sfl/Fle/Sw)	0.231	0.000	0.308	0.308	0.154
Sp	0.000	0.955	0.000	0.023	0.023
S (Sch/Sb/Sm)	0.051	0.615	0.000	0.000	0.333
F(Fm/Fst)	0.036	0.393	0.071	0.500	0.000
iii)	Facies stationary distribution $(\psi {P}_{ij}U$)				
All	0.108	0.400	0.152	0.206	0.134

**Figure 3:**
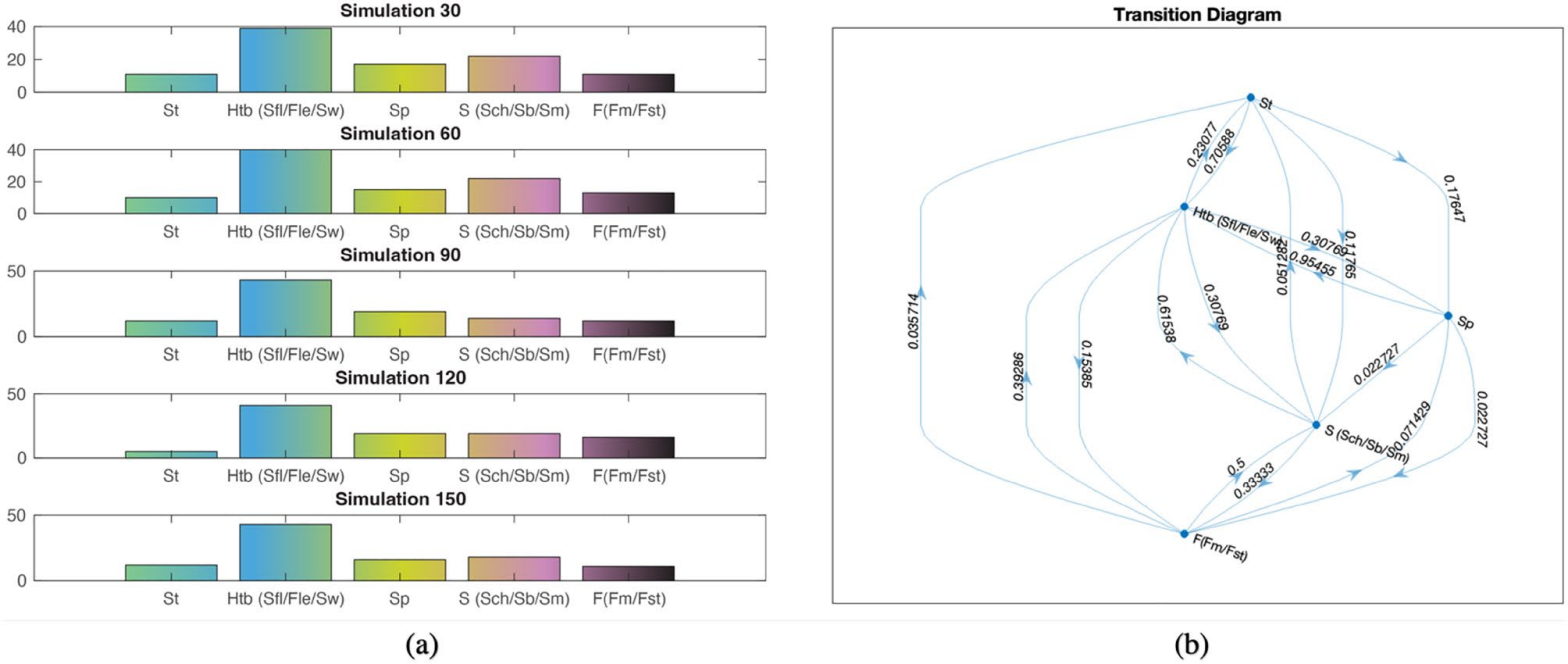
(**a**) Five sequential bar Markov results for the Surma group vertical facies transition where the dominance of each facies has been shown; (**b**) Transitional diagram or facies relationship diagram (FRD) of each lithofacies.

The stationary distribution demonstrated that at any random event, there is a ~ 40% chance of encountering heterolithic beds in the Surma group (Fig. 3a). The percentages for trough cross and parallel laminated sandstone are also high albeit they seem to be reduced due to multiple sand-shale alterations. This scenario can be well noticed in the transitional diagram and Markov bar results where the lithofacies relationship with each other has been summarized (Fig. 3b).

**Figure 4:**
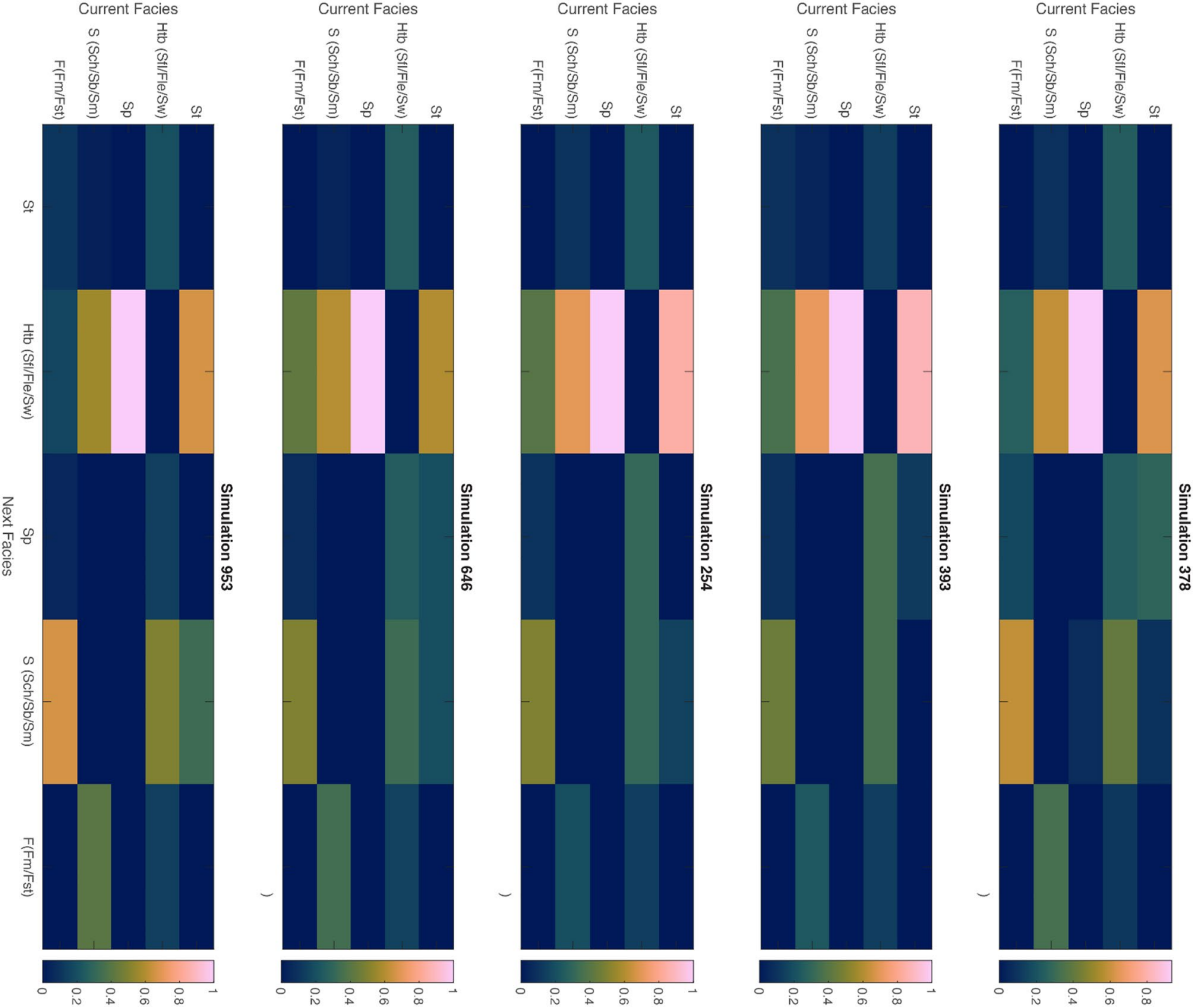
Generalized Markov chain simulation of current facies and the possibility of the next facies transition within the Surma group at any random event within the thousand models. The simulation has been generated based on the facies transition probability matrix followed by a defined initial state and sequence length.

We also simulated each of the facies based on the simple Markovian property. For a sequence length of 100 and 1000th simulation, there is significance observed in the Htb (Sfl/Fle/Sw) based on each defined current facies (Fig. 4). For instance, in simulation 254, the possibility of the occurrence Htb and Sp is approximately 70% and 25% after St as the current facies whereas in simulation 953 it is around 65% and 30% respectively. At any random simulation, flaser, wavy and lenticular bedding shows dominance in the vertical facies transition within the Surma group especially after encountering trough cross and planner laminated sandstone.

### Depositional settings

#### Wave-enhanced tide-dominated shallow marine environment with intermittent storm events

##### Description

**Figure 5:**
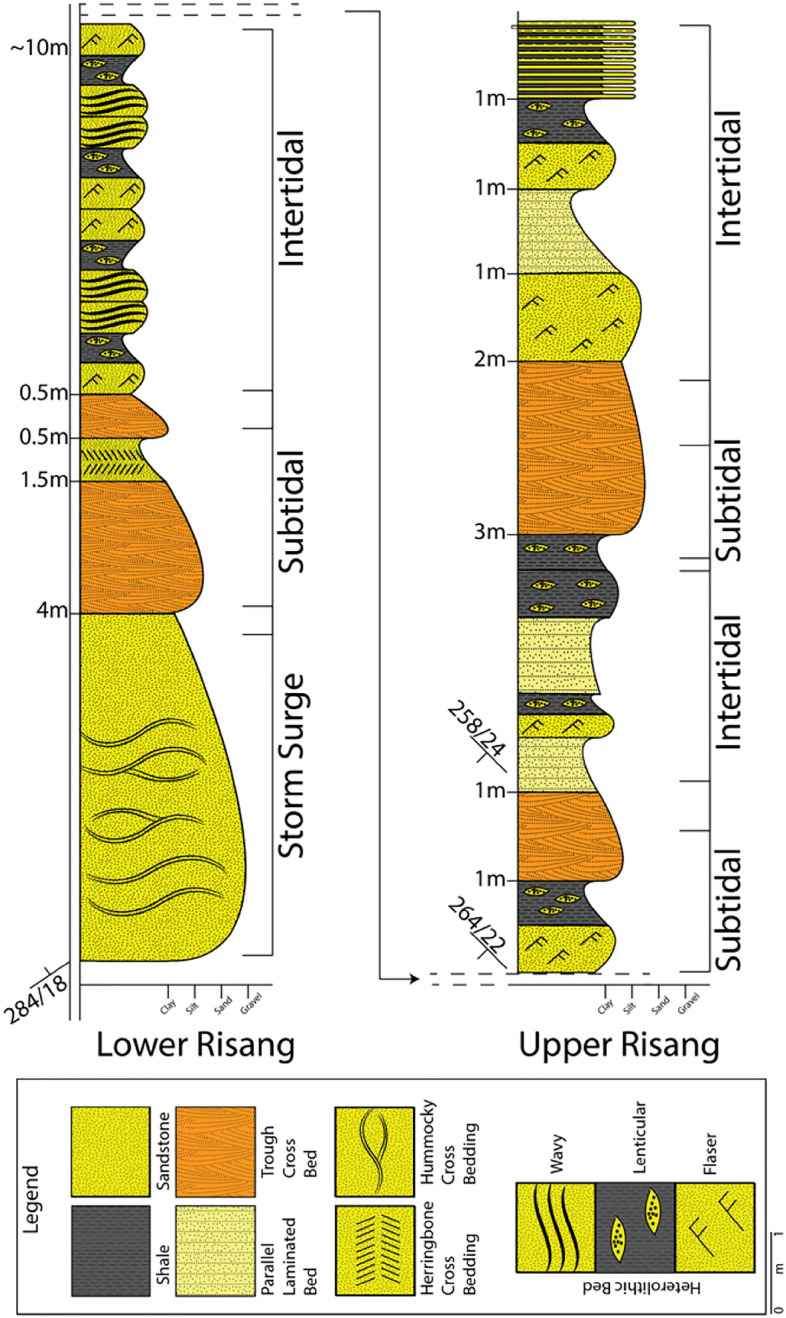
The lithological column of studied sections of Risang waterfall shows the Upper and Lower Risang with the subtidal and intertidal environmental sequences.

The facies association A_1_ can be divided into two distinct sedimentary facies assemblages (A_1_a- A_1_b) in the Risang waterfall section. The upper section is embedded with bluish-gray shale (Fissile Shale), heterolithic bed (HB), medium-grained and porous yellowish-brown sandstone, and clay-interbedded sandstone. Sedimentary structures such as trough cross-bedding (TCB), parallel lamination, flaser bedding, and lenticular bedding have been observed (Fig. 5). The orientation of the lithology was westerly dipping, and the amount of dip was between 20° and 28°.

The lower section is relatively sand dominated and has been exposed excellently where hummocky cross-stratification (HCS), herringbone cross-bedding (HCB), trough cross-bedding and ~ 20 cycles of heterolithic bed (Flaser, wavy and lenticular) was observed. The rock in this section is medium to fine-grained, yellowish gray in color, moderately hard, and has higher porosity due to lack of cement.

##### Interpretation

**Figure 6:**
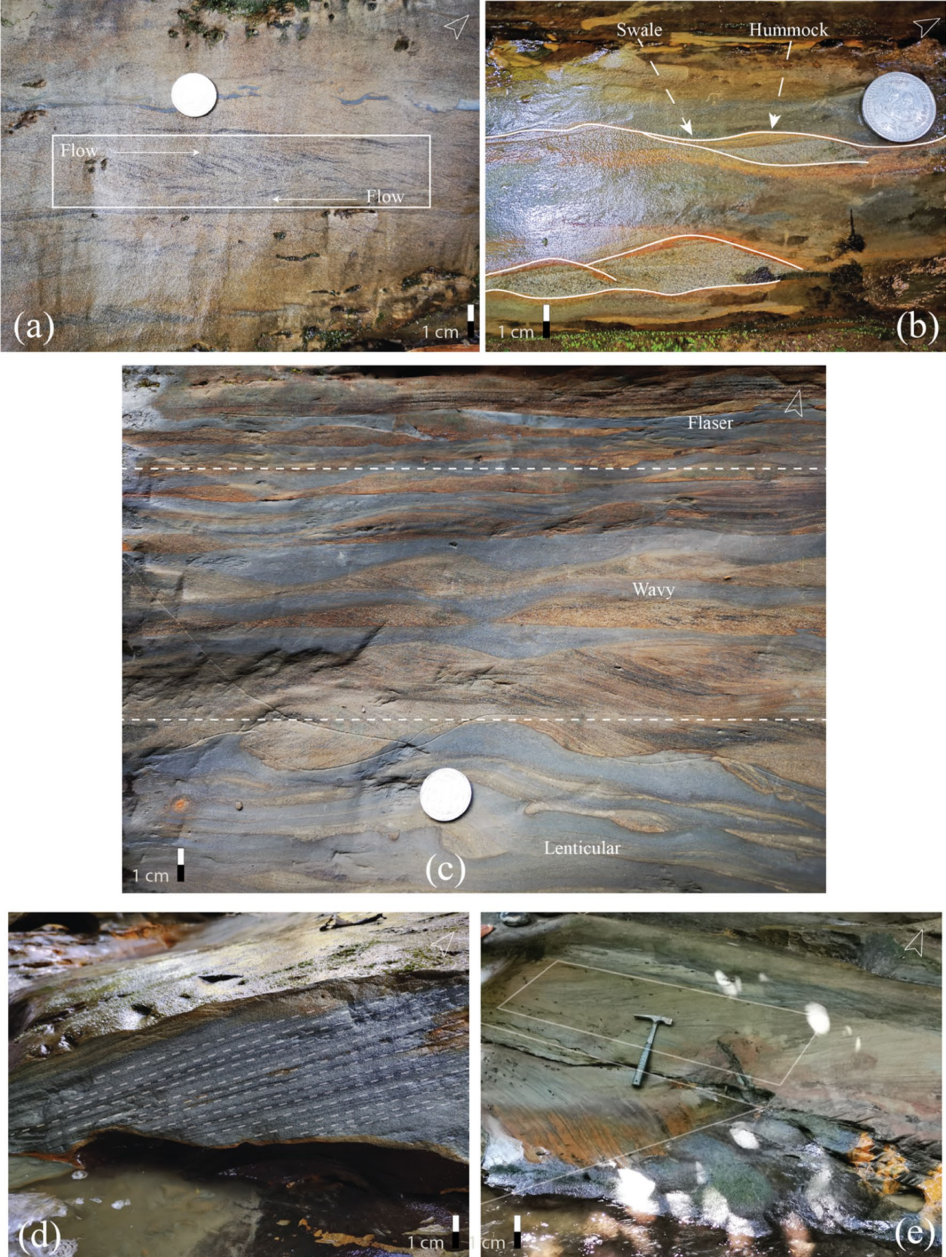
Different sedimentary features at Risang Waterfall section (Lower). The orientations have been documented in the lithological column. (**a**) Herringbone cross-stratification showing the bi-directional flow; (**b**) Hummocky cross-stratification demonstrating the swale (concave-up) and hummock (convex-up); (**c**) Heterolithic bed—lenticular (sand lenses withing clay), wavy (proportional sand and clay), flaser (clay drapes within sand); (**d**) Parallel laminated bedding; (**e**) Trough cross-bedding (The designated area is the area of truncation).

A fine-grained, greyish-white lithological unit with moderately compacted herringbone structure indicates a tidal environment with bi-directional flow (Fig. 6a). Fine-grained whitish sandstone containing hummocky cross-stratification indicates storm surge events (Fig. 6b). The confluence of unidirectional and oscillatory flow from HCB results from the action of relatively large storm waves in the ocean. Herringbone structure was formed when current periodically flowed in the opposite direction^10,52,53^.

Trough cross-bedding contends with whitish gray and fine to medium sand. TCB is evidence of higher energy (subtidal) conditions during deposition, whereas flaser, wavy and lenticular were comprehended when the energy became moderate and unstable (intertidal) during deposition time (Fig. 6c). Following up TCB, fine to medium-grained, yellowish-grey to white planner cross-bedded sandstone or parallel laminated bed encountered, which indicates the energy became weak (Fig. 6d,e). Finer particles accumulated on top of a bed as energy weakened (supratidal). However, the continuous repetition of HB and TCB construes that the section had undergone subtidal and intertidal deposits most of the time before wave-dominated deposits.

#### Subtidal to intertidal setting transitioning to channel deposits

##### Description

Apart from the repeated outcrop appearance of fissile shale, laminated sandstone, and massive claystone, the most common rock types are mudstone, and clay interbedded sandstone, followed by the existence of conglomerate, nodular shale, onion structure, and exfoliation nature of rocks. In Thakurchara, a significant litho-section can be seen, implying that it is analogous to an equivalent shale unit. The dip amount gradually decreased from the initial section (22°–12°).

##### Interpretation

Dirty white, very fine-grained claystone indicates the reductions in energy condition, which is corroborated by parallel laminated strata. The presence of laminated layers and dark-colored clay with very thin silty streaks in the outcrop suggests a minimal supratidal influence in the region, with the bulk indicating a subtidal-intertidal setting. Fine-grained, yellowish-white sand with silty streaks and siltstone was found, establishing the fining upward succession while the energy condition was lessened. The presence of sandstones with clay lenses indicates channel abundance, and the ex-situ conglomerate on the way of traversing showed the existence of channel lag deposits. Channel-lag deposits are coarse residual materials left as accumulations in the channel in the normal processes of the stream^54^. The examined flank is tectonically active, and the presence of exfoliation rocks, nodular structures, and the exfoliation nature of stones suggests the position of the anticlinal axis nearby^55^. Due to rolling tectonic activity and the influence of coupling forces over the opposite direction in an inflation point, various micro joints developed and an almost vertical bed during the traversing, hypothesizing the existence of a thrust fault in the western flank.

**Figure 7:**
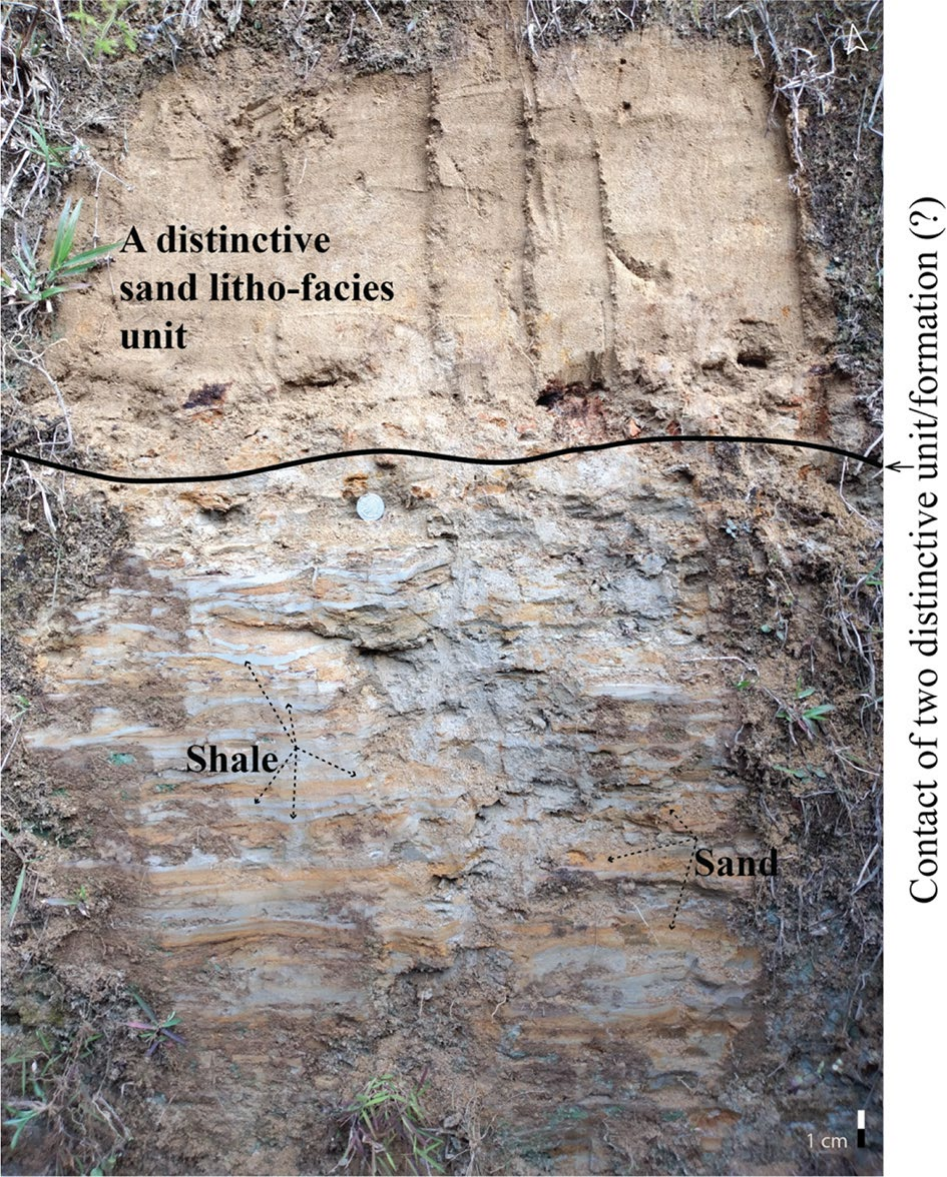
An equivalent shale facies (An Equivalence to Upper Marine Shale (?)). This feature indicated a zone of transition between higher and lower energy.

An equivalent shale facies with heterolithic beds comprehended with yellowish-brown color sand is believed to be a transition zone between high and low energy conditions (Fig. 7). Changing from a high-energy to a low-energy state does not occur spontaneously in the unique facies association. This equivalent shale unit is hypothetically the transition zone between two distinctive litho-units (formation).

#### Interplay of fluvio-deltaic distributary settings with possible diagenetic process

##### Description

**Figure 8:**
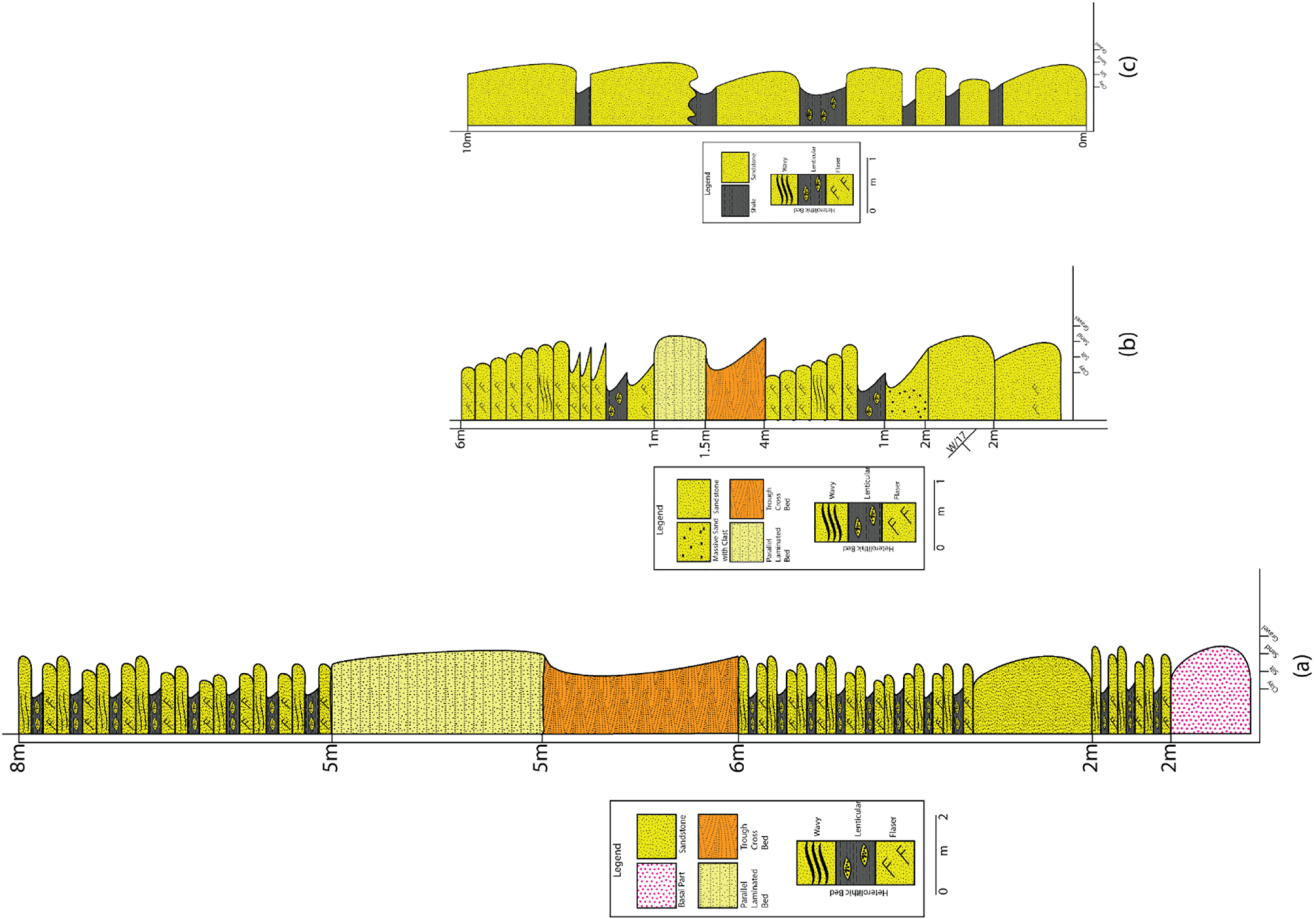
(**a**) Entire Alutila Cave section (partial); (**b**) Inside the cave section; (**c**) Alutila army camp section.

The Alutila cave's basal part constitutes coarse-grained sandstone that is moderately friable and has an approximate porosity of 15–20%. The span of the entire cave section (bottom to top) contends with a heterolithic bed, massive sandstone, trough cross-bedding, and parallel laminated bed (Fig. 8a). In the cave, massive sandstones are filled up with clast and leaching due to their excellent permeability, the zone of weakness (Fig. 8b). Structures encountered in the sections are load cast, climbing ripples, flame structure, erosional base, micro cross lamination, convolution, and vertical joints. Sand-shale alternation lithology and yellowish-brown coarser sandstone have also been encountered outside of the cave’s section. In addition, the army camp section is embedded with vertically stacked channels reuse that is resembled by sand injectites and load cast owed by channel fill deposits (Fig. 8c).

##### Interpretation

**Figure 9:**
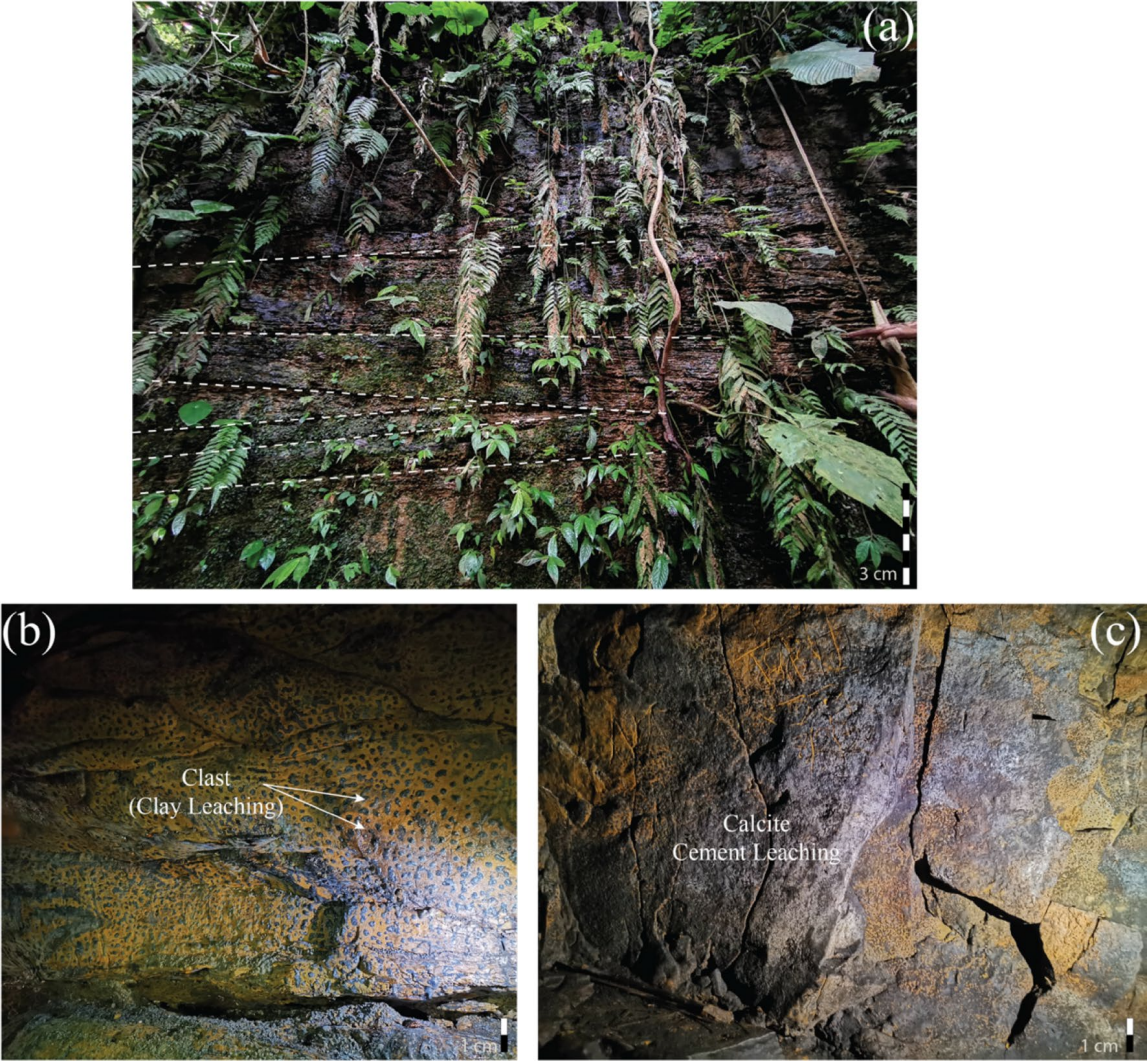
Different features of Alutila cave: (**a**) Outside section of Alutila cave where series of trough cross bedding has been remarked; (**b**) Clay leaching in massive sandstone which might be confused with the in-situ conglomerate; (**c**) Calcite cement leaching, which was immediately remarked on the spot (small-scale) with the chemical effervescence.

In addition to fine-grained and structureless massive sandstone, trough and parallel cross-bedding were quite frequent (Fig. 9a). Several unidentified clasts were visible on the surface of the massive sandstone (Fig. 9b). These clasts were possibly formed due to the leaching activity of clay and other cementing materials, which emphasize higher porosity and permeability in massive sandstone^56,57^. TCB and PCB consequently showed high and lower energy conditions, followed by the heterolithic bed. Sand injected in clay comprehended a cause of the failure of shale permeability fie^58–60^.

The most common formation of caves is developed by the dissolution of limestone^61^. Rainwater has the propensity to acquire carbon dioxide from the surrounding atmosphere, and as it infiltrates the soil, it undergoes a transformation into a dilute acid. The process at hand gradually facilitates the dissolution of limestone along the joints, bedding planes, and fractures, resulting in the enlargement of certain crevices to the extent that they manifest as caves^61,62^. Albeit a limestone cave is typical, a cave formed through sandstone is rare^63,64^. Alutila is an unusual place for large cave formation, triggered by several geological factors, i.e., uniform lithology; zone of weakness; and adjacent higher gradient.

Massive sandstone has relatively higher porosity (φ) and is less resistant than heterolithic bed^65,66^. So, any leaching activity can easily pass through it. But large-scale calcite cement wasn’t encountered, which could be dissolute the cave. Instead, some cementing material formed the cave roof and wall clasts (Fig. 9c). The number one factor that might have initiated the cave formation is large-scale joints and fractures exposed to the surface. These joints and fractures are called the zone of weakness, along which water could percolate easily and erode massive sand bodies. As this is a uniform lithology (i.e., no shale barrier over sand), stream flow easily cuts through these sand bodies. The relief difference between the entrance and exit makes a high gradient and energy to wash out the uniform lithology more profoundly. The cave didn’t collapse due to concretion and a calcareous band over the roof, which is very resistant. The erosional activity is confined within the massive SST. Hence, we can concur that the cave is entirely lithology-controlled.

#### Complex marine-deltaic setting to transgression event and fluvial environment

##### Description

**Figure 10:**
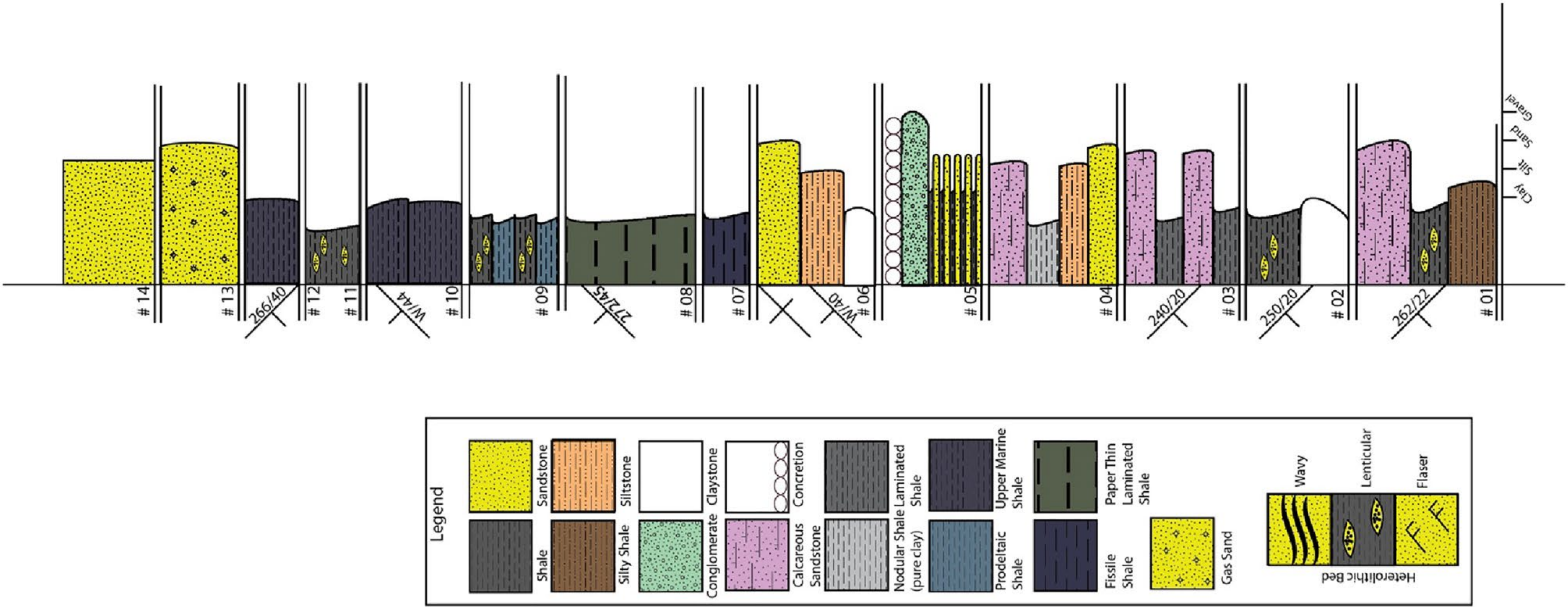
Discrete lithological column of the Dhoilachara-Bangmara section (Scale was not given for the visual representation as this section was conducted under ungetatable conditions; later described the section uniformly and coherently).

The reconnaissance of the Dhoilachara-Bangmara subsection was performed discreetly due to the inaccessibility throughout the investigated region. Claystone, siltstone, mudstone, massive sandstone, calcareous sandstone, fissile shale, laminated shale, nodular shale, pro-deltaic shale, heterolithic bed, conglomerate, and concretion was predominant litho-facies of this sub-section (Fig. 10). In addition, paper-thin laminated shale was unique facies encountered in the section. It was also discerned that channel lag deposits and vertical channel stacking were occurring on a limited scale. Structures such as drag fold, crisscross joint, load cast, ripple marks, hummocky cross-stratification, and parallel lamination were encountered.

A predominant shale unit was encountered, in which lateral extension distinctly divided the other two litho-facies association units. The former shale unit is highly jointed, thinly laminated with the occasional silty streak, and consists of unidentified flaky clay minerals. The latter is a yellowish brown, and the coarser sand unit and gas sand were also distinguished underlain on this sand unit. The leaching has transformed the yellowish-brown color into a relatively bluish-gray.

##### Interpretation

Whitish grey and very compacted fine-grained calcareous sandstones were found, which might have formed due to the leaching activity of calcite cement in sandstones^67,68^. In contrast to the conglomerate found in the Risang-Thakurchara section, the conglomerate confronted here along the traversing path is in-situ, which was more likely to be para-conglomerate which was not uniform in thickness. The tidal influence was indicated by the frequent heterolithic bed. Nevertheless, fine-grained, yellowish-white sand with a silty streak and siltstone successively advocated the forms of the fining upward sequence and were supported by very fine-grained mudstone, which suggests the lowering energy state^10,53^. Several sub-lithofacies of shale, i.e., paper-thin laminated shale, fissile shale, nodular shale, and pro-deltaic shale are also evident in the reduction of tidal influence^52,69^. The well-exposed bluish-grey shales contain parallel lamination structures, flaky clay minerals, and silty streaks. These facies indicate a small-scale marine transgression event^70^.

The presence of gas sand and the leaching of Fe also imply an excellent porosity and permeability of these facies. This unit has every parameter of being a good reservoir except a barrier which makes it ineffective for reserving any gas. Structures, i.e., drag fold and crisscross joint also prescribed the section as a tectonically active region. In addition, encountering a high dipping vertical bed implies that this area underwent the experience of thrust faulting. We also found distinct yellowish-brown hue, fine to medium coarse grain, and loose to medially compacted sandstone, indicating a bar-deposited fluvial system^29^. However, these facies (and associated units) weren’t studied comprehensively during the investigation.

#### Tidal-influenced channelized fluvio-deltaic setting with erosional activity

##### Description

**Figure 11:**
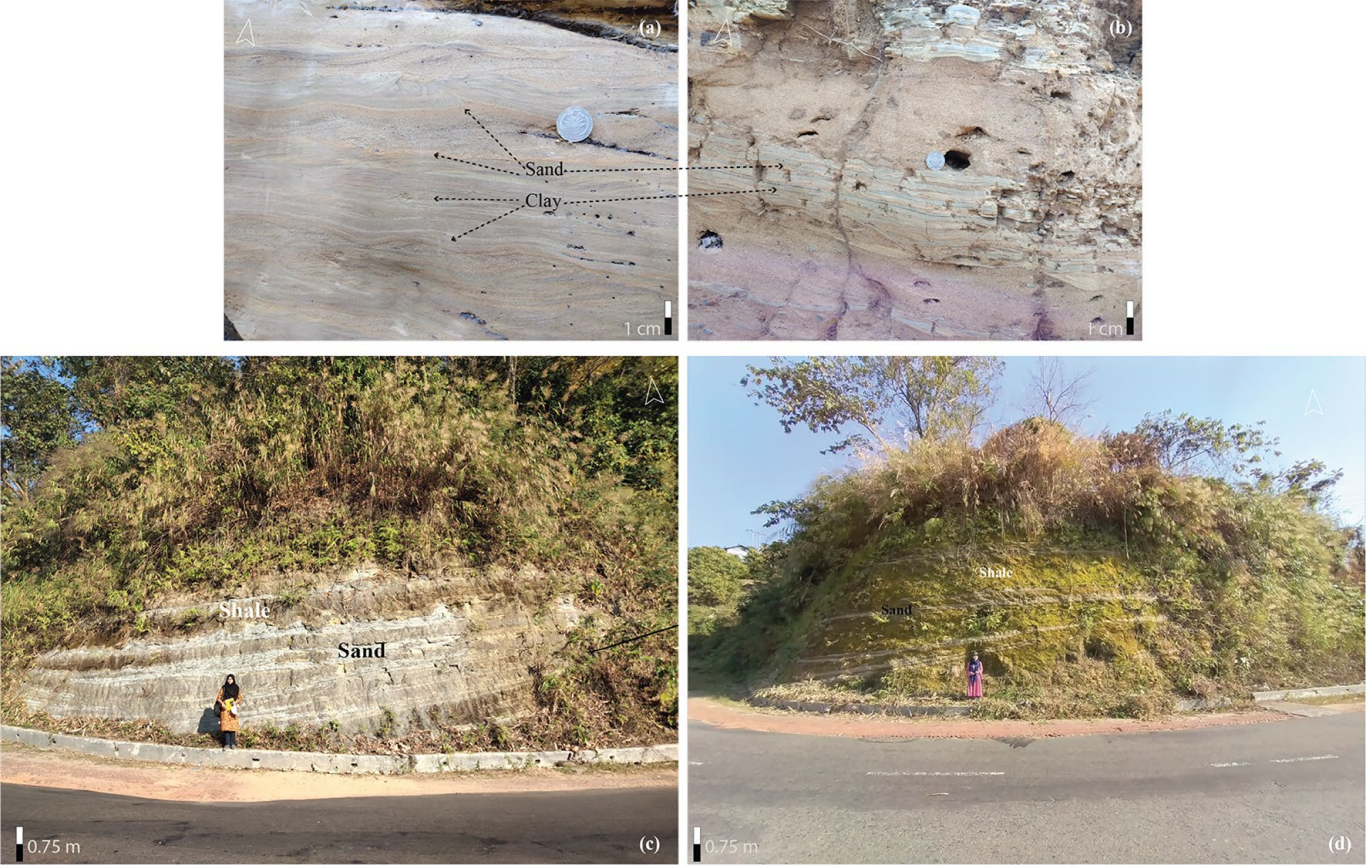
Studied outcrop in the Matiranga-Alutila road cut section: (**a**) Ripple laminated sand (Also small scale convolution seen in the upper part); (**b**) Heterolithic bed; (**c**)–(**d**) Sand injected in the vertically stacked channel body.

Heterolithic bed, trough cross bed, ripple laminated sand, parallel laminated bed, channel sand, stacked channel, and injectites were the predominant litho-facies along the road cut section (Fig. 11a, b). Micro cross lamination, flame structure, and convolution structures were also observed besides gradation (which was not so prominent). Leaching has been identified as a contact of sand and silt facies, whereas some heterolithic beds underwent highly weathering materials. Large-scale and laterally extensive channel sand was also delineated in the section.

##### Interpretation

Albeit heterolithic beds faced significant weathering events, TCB and paralleled laminated sandstone indicate tidal fluctuations^7,53^. However, graded bedding suggests that somewhat channel-fill deposition might have occurred during tidal fluctuation^71^. Channel fill clay and soft sediment deformation indicate the possibility of lateral migration history. A vast channel sand (amalgamated channel sand) has been identified by the geometric pinching on its lateral sidebar as a landing base if extensive erosion occurs (Supplementary Information Figure S8). This sand body has been deposited under the influence of a stream bed and experienced erosional activities in the underlying bed^50,72^. Sand Injectites are a type of sedimentary body where sand injects into soft clay. It is embellished with higher porosity and permeability, making it an excellent reservoir^59^. The intrusion of sand has multiple degrees of volumetrization of carrying hydrocarbon. In the Matiranga-Alutila road cut section and Alutila Army camp, several injectites bodies have been encountered (Fig. 11c, d).

## Discussion and conclusion

The deposition of Miocene sediment in the Bengal Basin is widely speculated to have occurred within a deltaic to shallow marine setting^29,50^. Nevertheless, other studies in CTFB also suggest the possibility of deep marine turbidities deposits^21,35,70^. In this study, Unit I is the representation of the Upper Surma group (corresponding to the conventional Bokabil formation) that comprises alternation sandstone and shale with minor siltstone and occasional conglomerate deposits^10,17^. Unit II is a marine shale sequence that is deposited under the influence of oceanic conditions. Whereas the previous unit suggests regression events, this unit (Upper Maine Shale) offers a small-scale transgression event in the Bengal Basin on a regional scale^70,73^. It is unlikely that the energy condition fluctuates from one condition to another spontaneously without a transitional zone. This unit (Fms/Fms eq.) is hypothetically that transition zone which has not been established yet comprehensively except synthetically field observation and seismic reflector^20,70^. Unit III (Tipam Group) is sand-dominating, and the characteristics of color and texture clearly distinguished this unit from previous sandstone depositional conditions^17,24^ (Fig. 2).

**Figure 12:**
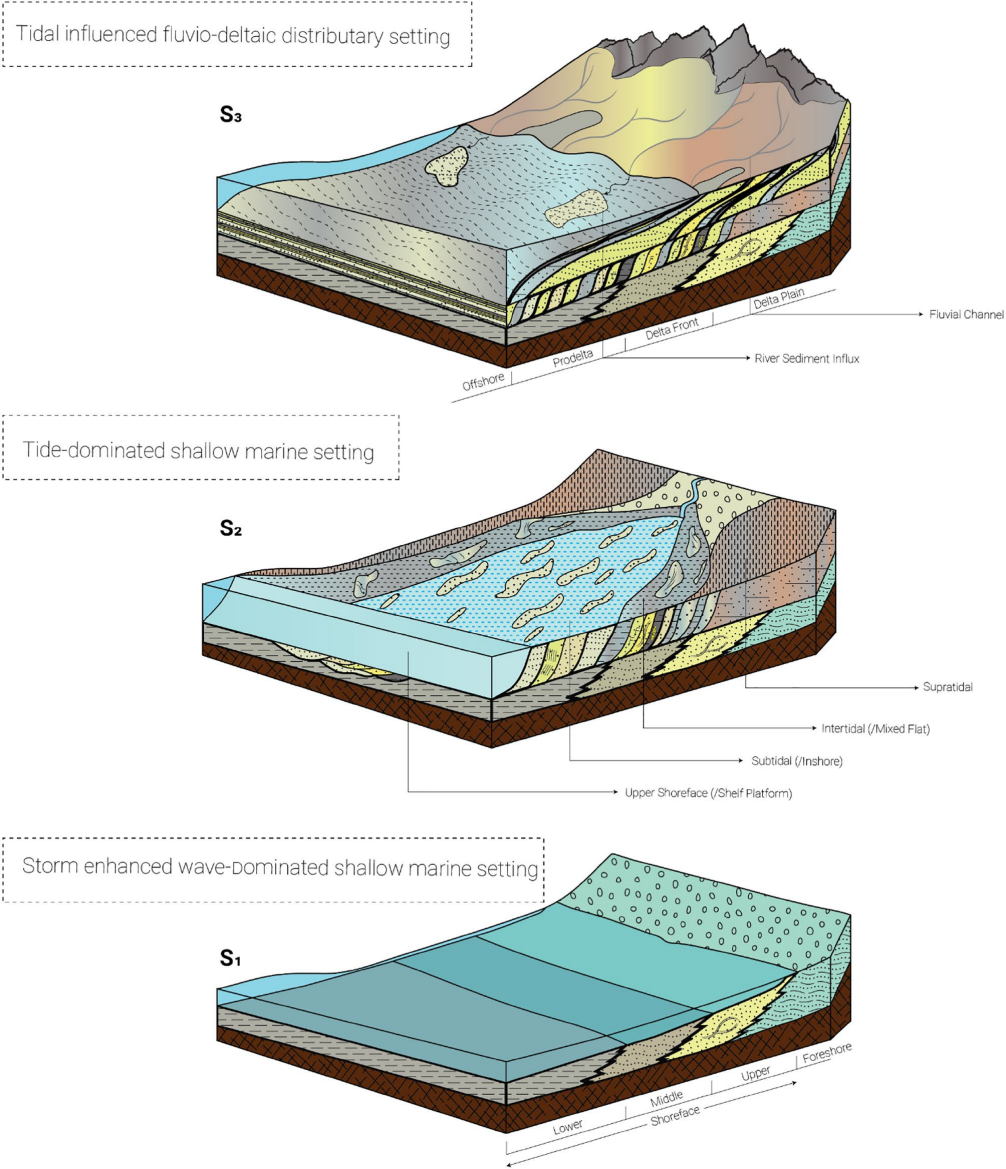
Schematic three-stage depositional settings for the Upper Surma Group at CTFB region in Bengal Basin. S1 (Older) to S3 (Younger) represent the environment from wave-dominated shallow marine settings to fluvio-deltaic distributary settings with tidal influences at each stage^78,84,85^.

Establishing the distinct heterogeneity between the conventional Bhuban and Bokabil formations poses a significant challenge due to frequent lateral facies changes and vertical variations of the facies. A paleoenvironmental evolutional history can be discussed for the Upper Surma based on the highly calibrated outcrop-based sedimentary logs for facies analysis and Markovian analysis (Fig. 4). We propose a three-stage conceptual depositional model for the Upper Surma Group addressing facies associations with their respective architectural elements, structures, spatiotemporal distribution, and transition probability (Fig. 12). While it is necessary to include both thorough quantitative and qualitative approaches to model depositional elements, our study area of Changotaung is considerably remote and not easy to access to frequently depict all the elements.

Hummocky cross-stratification (HCS) was quite frequent at the base of the Risang Waterfall section. This hummock and associated swale are one of the prime indicators of frequent storm surges that apparently occur in the shallower part of the marine settings^74^. It is quite challenging to differentiate between the shorefaces, particularly the transition between the middle and lower shorefaces. Notwithstanding this, HCS deposits are most commonly found at the transition zone between the lower shoreface and the foreshore (shelf) where wave interaction with the seafloor sediment takes place during storm events^75–78^. Although similar hummocky cross-stratification has been discerned in the other CTFB structures, there is still no documentation yet regarding exposed HCS in the Sylhet trough^52,79^.

Based on the simple first-order modified EMC analysis, the dominant frequency of the heterolithic beds has been deciphered after encountering different lithofacies within a vertical facies transition. At each step, more than 40% probability of Htb indicates the repetition of flaser, lenticular and wavy bedding followed by trough cross-bedding and parallel laminated bedding. Trough crossbedding provides indicative of subtidal circumstances during deposition while flaser, wavy, and lenticular were comprehended when energy became intermediate and prone to fluctuating^78,80^. We tried to decipher frequent tidal signatures and elements throughout the exposure and facies associations. Hence, we didn’t discuss the sub-environment settings of tide-dominated settings such as tidal mud flat, tidal sand flat, or tidal sand bar. However, while flaser and wavy bedding proposed tidal sand bar facies, lenticular and wavy demonstrated tidal mud flat facies construction^78^. The erosion of the mud and depositions of the sand determines the dominant environment with the current speed, yet the bi-directional current flow is common in the sandy portion^81–83^. The flow might have also influenced the fluvial deposition at multiple spatiotemporal positions. For instance, massive sandstone, channel lag deposits, and channel sand are frequent in both the CTFB and Sylhet trough of the Bengal Basin^10,52,79^. The origin history of Alutila Cave including unidentified clast-supported sandstones, erosional base, and lenticular bodies supports fluvial deposits of late Miocene time. Because of the presence of a distant research region with limited accessibility, as well as disparities in elevation, and ambiguity about the axis of the overall structure, the irregular progression towards finer sedimentation in the channel appearance was not discerned here.

Earthquake-induced deformation in the strata observed in both modern and ancient sedimentary succession can shed light on the paleoseismic events contributing to post-depositional processes within a specific diachronous unit^86–88^. Even though we encountered soft-sediment depositional (SSD) structures such as convolution, and small-scale load cast, it is unlikely that it comprehends the full post-depositional history for Miocene sediment. Drag fold and sands forced injects into clay (injectites) may be related to as intense deformation and uplift history of the Bengal Basin during the Late Miocene-Pliocene timeframe^89–91^. Both liquefied features and compressional features identified in the Upper Surma group suggest that it experienced both high sedimentation load and earthquake-triggered deformation activities during its meta and post-depositional periods^14,91,92^.

We believe the Upper Surma Group has experienced three interconnected different depositional environments in the Chittagong Tripura fold belt region while it mostly experienced fluvio-deltaic distributary settings in the Sylhet trough. This constrains the traditional belief of the Surma group's (Upper) depositional pattern and can be used to study the revision of the stratigraphic scheme of the Bengal Basin including syn, meta, or post-depositional history. The hypotheses should be extensively tested together both in the Sylhet trough and other CTFB structures for a better stratigraphic scheme, depositional and tectonic history. Changotaung structure is quite remote to date compared to other CTFB structures and hence, there is some limitation in this study including the Markovian analysis. Albeit our current study is restricted within a certain region, it contributes to the current understanding of the sedimentary system of the Chittagong Tripura Fold Belt (CTFB) and paves the way to establish a framework for stratigraphic rock records of the late Cenozoic era.

### Supplementary Information

Supplementary Information 1.

Supplementary Information 2.

Supplementary Information 3.

Supplementary Information 4.

## Data Availability

All data generated or analyzed during this study are included in this published article and its supplementary information files. The scripts can be accessed within the StratigraphicMM GitHub repository (https://github.com/saifulapu/StratigraphicMM.git). Rivers and drainage data used for map generation can be found at https://data.humdata.org/. Some basic petrographic data sets and analyses are available from the corresponding author upon reasonable request.
