# Supplementary figures and images for: High-resolution lithostratigraphy and reconnaissance sedimentology of Changotaung structure, Chittagong Tripura fold belt, Bengal Basin, Bangladesh
Source: Sci Rep. 2023 Oct 18;13:17727. doi: 10.1038/s41598-023-43810-7 (PMC10584892; doi:10.1038/s41598-023-43810-7)

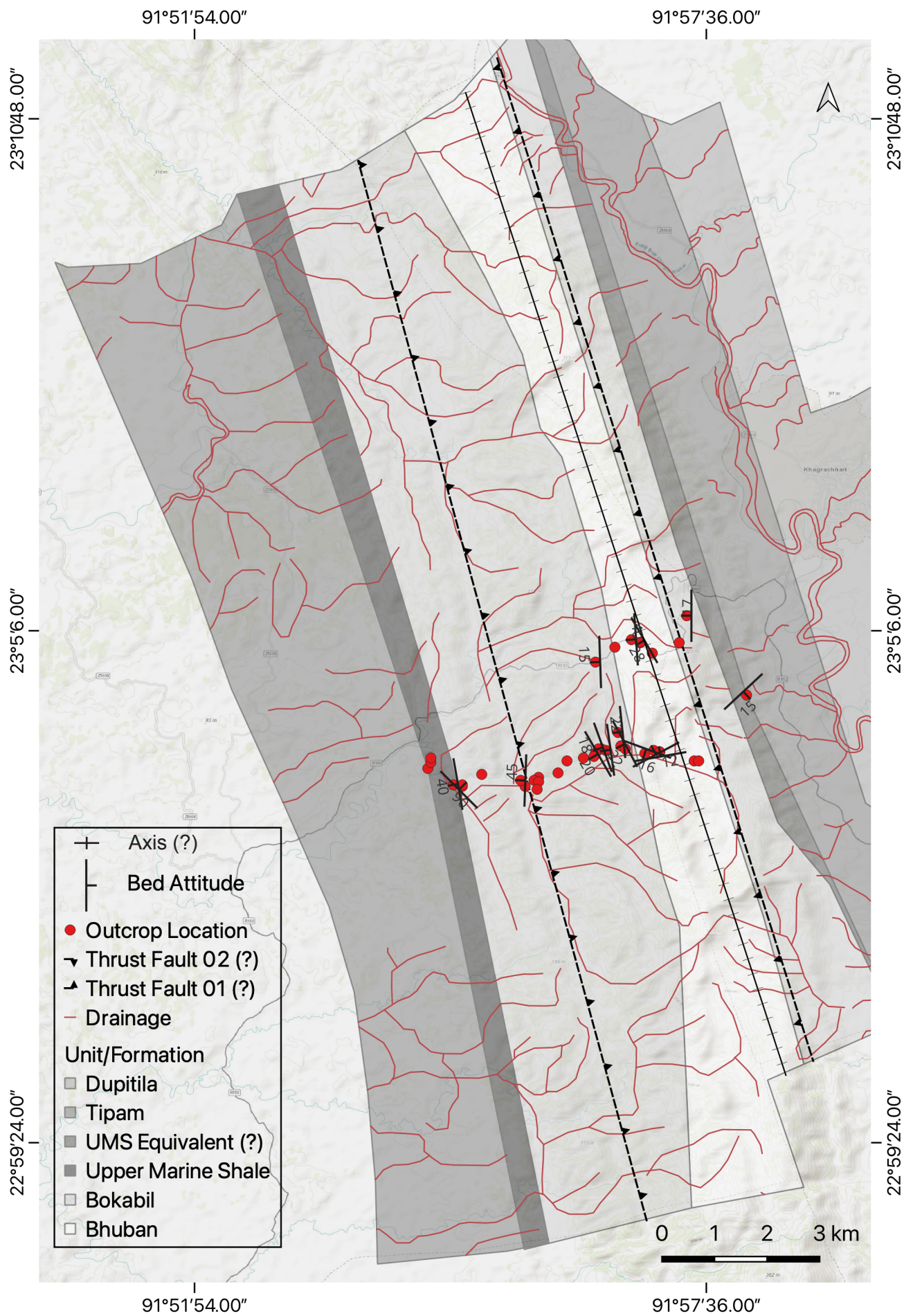

Supplement: Supplementary file 1 — Supplementary Information 1. [file 41598_2023_43810_MOESM1_ESM.pdf]
