# Supplementary material for: High-resolution lithostratigraphy and reconnaissance sedimentology of Changotaung structure, Chittagong Tripura fold belt, Bengal Basin, Bangladesh
Source: Sci Rep. 2023 Oct 18;13:17727. doi: 10.1038/s41598-023-43810-7 (PMC10584892; doi:10.1038/s41598-023-43810-7)
Supplement: Supplementary file 2 — Supplementary Information 2. [file 41598_2023_43810_MOESM2_ESM.pdf]

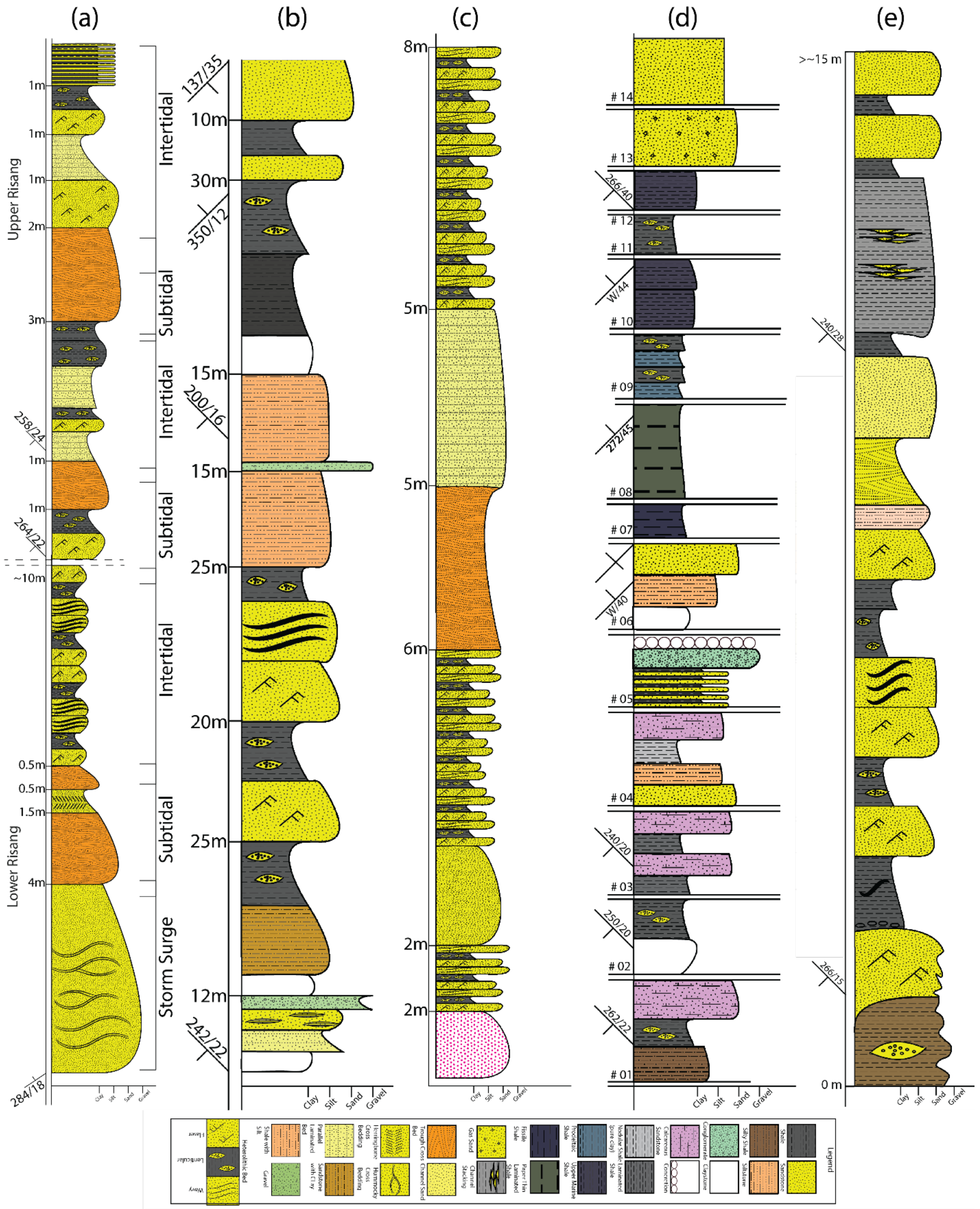

Summarized lithological column of (a) Studied sections of Risang waterfall showing the Upper and Lower Risang; (b) Risang-Thakurchara section; (c) Entire Alutila Cave section; (d) Discrete lithological column of the Dhoilachara-Bangmara section; (e) Matiranga-Alutila Road Cut section.
