# Supplementary material for: High-resolution lithostratigraphy and reconnaissance sedimentology of Changotaung structure, Chittagong Tripura fold belt, Bengal Basin, Bangladesh
Source: Sci Rep. 2023 Oct 18;13:17727. doi: 10.1038/s41598-023-43810-7 (PMC10584892; doi:10.1038/s41598-023-43810-7)
Supplement: Supplementary file 3 — Supplementary Information 3. [file 41598_2023_43810_MOESM3_ESM.pdf]

High-resolution Lithostratigraphy and Reconnaissance Sedimentology of Changotaung Structure, Chittagong Tripura Fold Belt, Bengal Basin, Bangladesh

[a,b]Noshin Sharmili, [a,c]Saiful Islam Apu, [a,d]Md. Yousuf Gazi, [a]Md. Anwar Hossain Bhuiyan*, [a]Janifar Hakim Lupin

[a]Department of Geology, University of Dhaka, Dhaka-1000, Bangladesh.
[b]Department of Geosciences, The Pennsylvania State University, University Park, PA-16802, United States.
[c]Department of Geology, The University of Kansas, Lawrence, KS-66045, United States.
[d]School of Geosciences, Faculty of Science, University of Sydney, Australia.
*Corresponding Author, Email: ahb@du.ac.bd

Supplementary Information Lists

The following supplementary information's are extra images and information additions to the main manuscripts.

S0 Sampling and reading location of Changotaung Anticline
S1 Investigated Section
S2 Bed Orientation

S0 Sampling and reading location Changotaung Anticline

The Alutila-Risang-Thakurchara and Risang-Dhoilachora-Bangmarasetu sections were used to explore the reconnaissance geological features and attributes of the Changotaung structure. The inquiry into specifics might be separated down further into its subsequent sub-section.

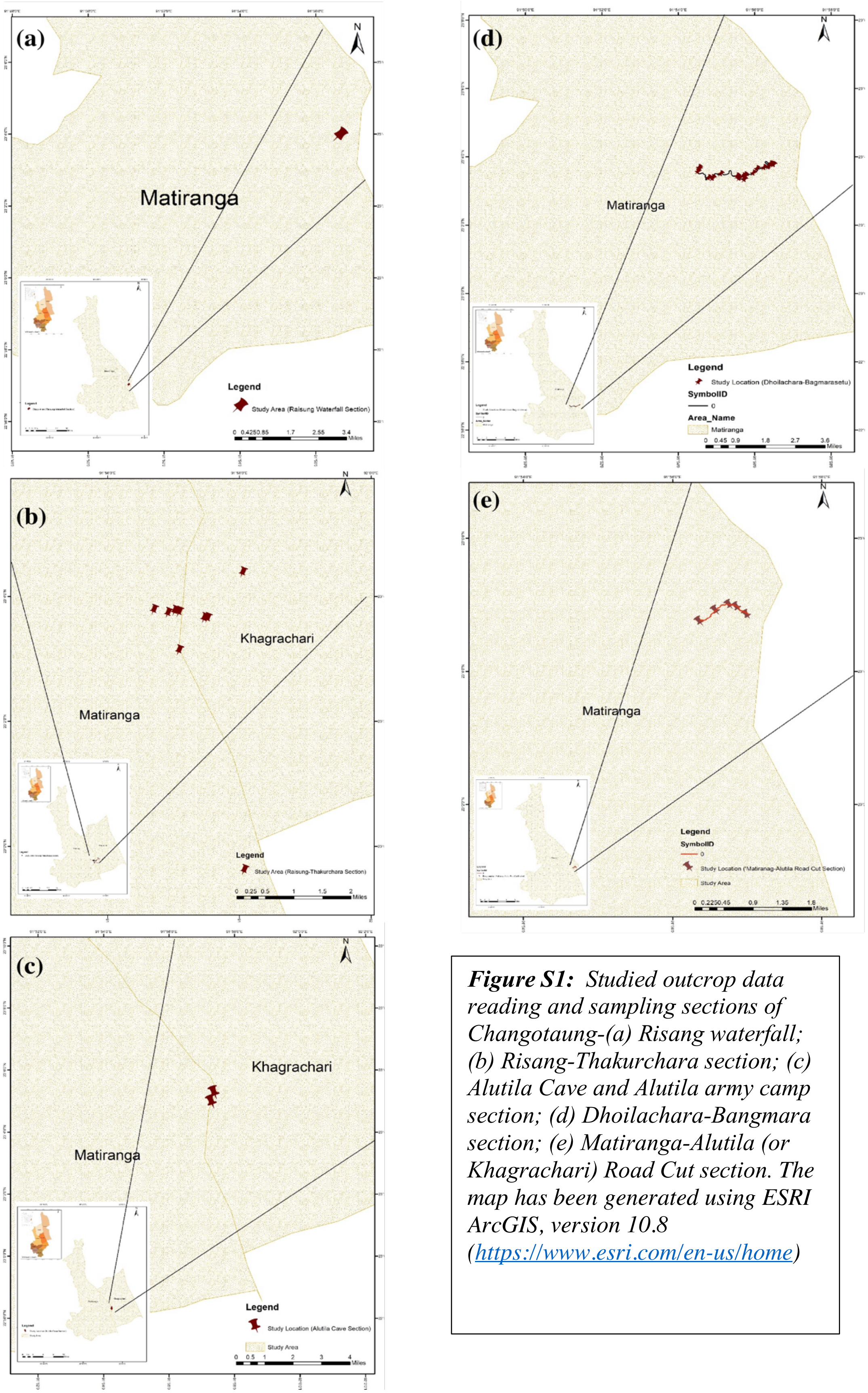

Figure S1: *Studied outcrop data reading and sampling sections of Changotaung-(a) Risang waterfall; (b) Risang-Thakurchara section; (c) Alutila Cave and Alutila army camp section; (d) Dhoilachara-Bangmara section; (e) Matiranga-Alutila (or Khagrachari) Road Cut section. The map has been generated using ESRI ArcGIS, version 10.8 (https://www.esri.com/en-us/home)*

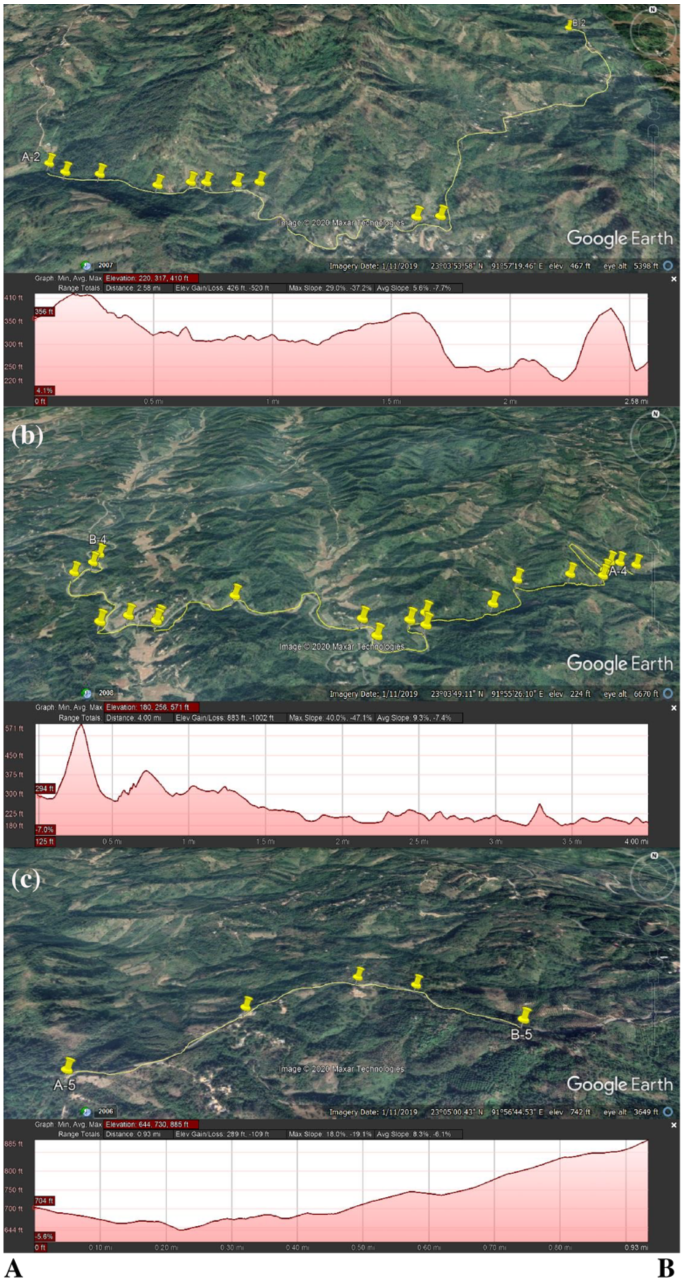

Figure S2: *KML image panel of the outcrop data reading and sampling sections of Changotaung-(a) Risang-Thakurchara section; (b) Dhoilachara-Bangmara section; (c) Matiranga-Alutila Road Cut section. Full panel view has been given below. The figure has been produced using Google Earth Pro (https://www.google.com/earth/versions/)*

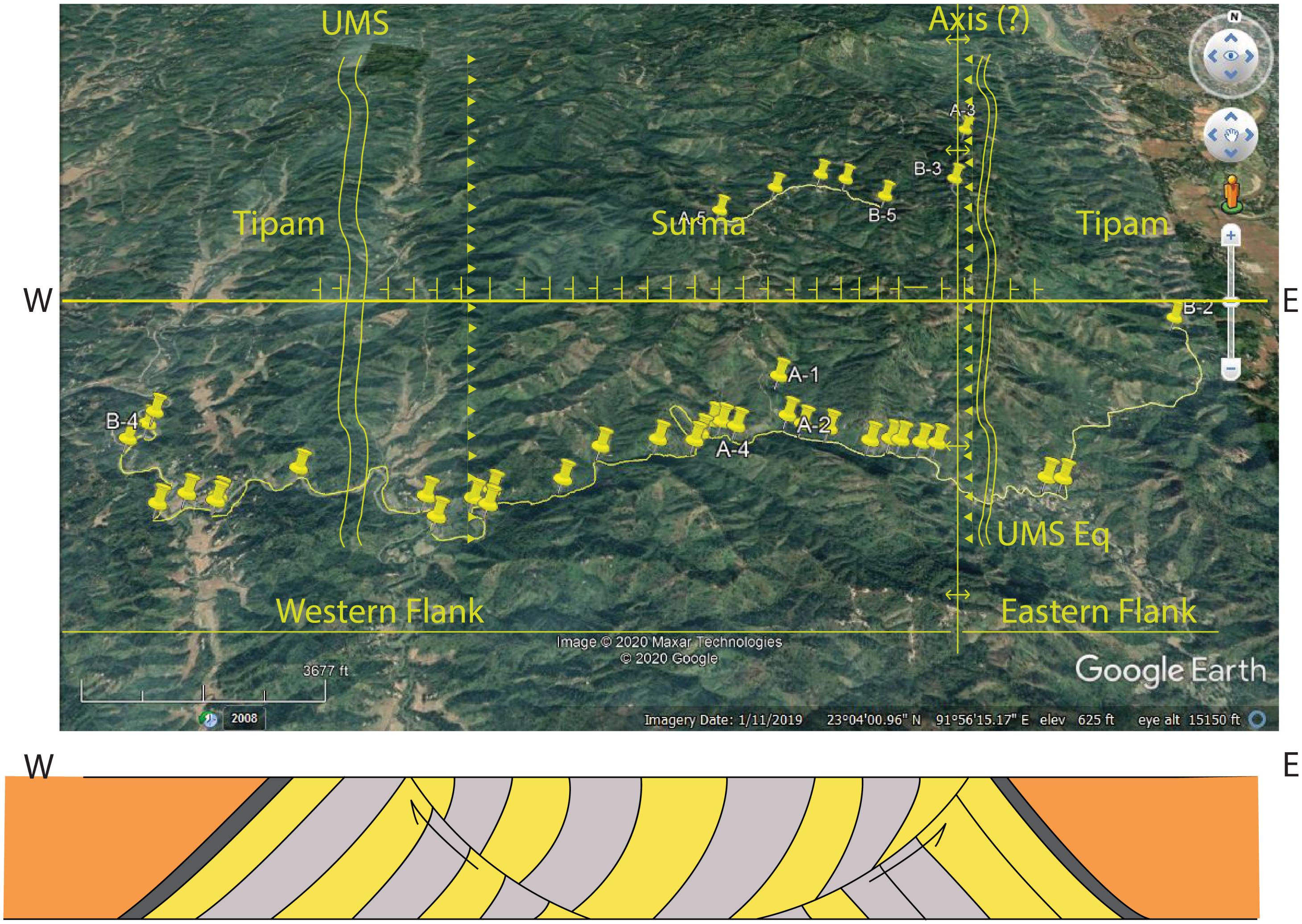

Figure S3: *Schematic Cross section of Changotaung Anticline illustrated (not drawn to the depth scales) based on the unit interpretation from the lithofacies analysis at different sections. The investigated sampling and measurement locations are also denoted with yellow marks.*

S1 Investigated Sections

a) **Risang Waterfall** (23.066111°N, 91.943611°E)

Risang Waterfall (aka Raisung) is ~3 Km west of Alutila tourism center (~1 km from Khagrachari-Chittagong road). This spring's natural fall is over 25 meters high.

Sedimentary features and facies: Bluish gray fissile shale; Heterolithic beds; Medium grained & porous yellowish-brown Sandstone, and clay interbedded sandstone; Trough cross-bedding, parallel lamination, Hummocky cross-stratification, herringbone cross-bedding.

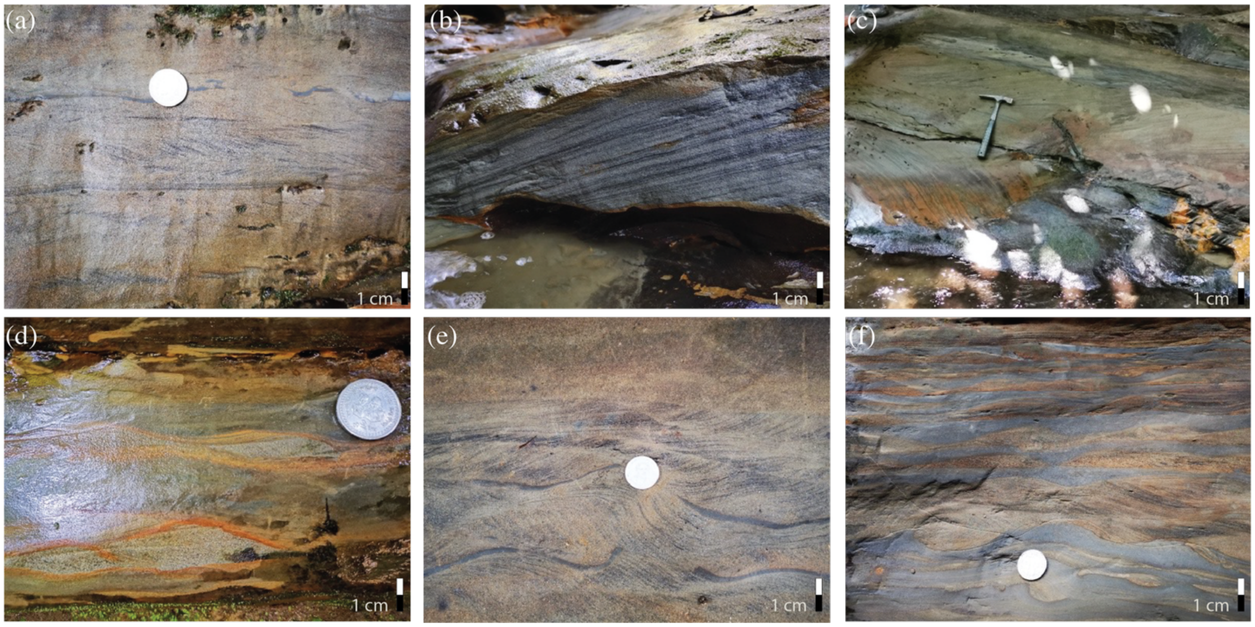

Figure S4: *Different sedimentary features and stratigraphic column of Risang waterfall section (a) Herringbone cross-bedding; (b) Parallel lamination; (c) Trough cross bedding; (d) Hummocky cross-stratification; (e) Convolution and flame structure; (f) Heterolithic beds.*

b) Risang-Thakurchara (23.063333°N, 91.944167°E - 23.073056°N, 91.975°E)

The length of this segment was over 7.5 km, and KML visual inspection revealed that its highest and lowest elevations were, respectively, 410 feet and 220 feet along the traverse path. The investigation has been carried out on the western flank and on the eastern flank, albeit only in small parts.

Sedimentary features and facies: Claystone and mudstone; Laminated sandstone; Fissile shale; Clay interbedded sandstone; Conglomerate; Nodular shale; Onion structure and exfoliation; Dark clay with silty streaks.

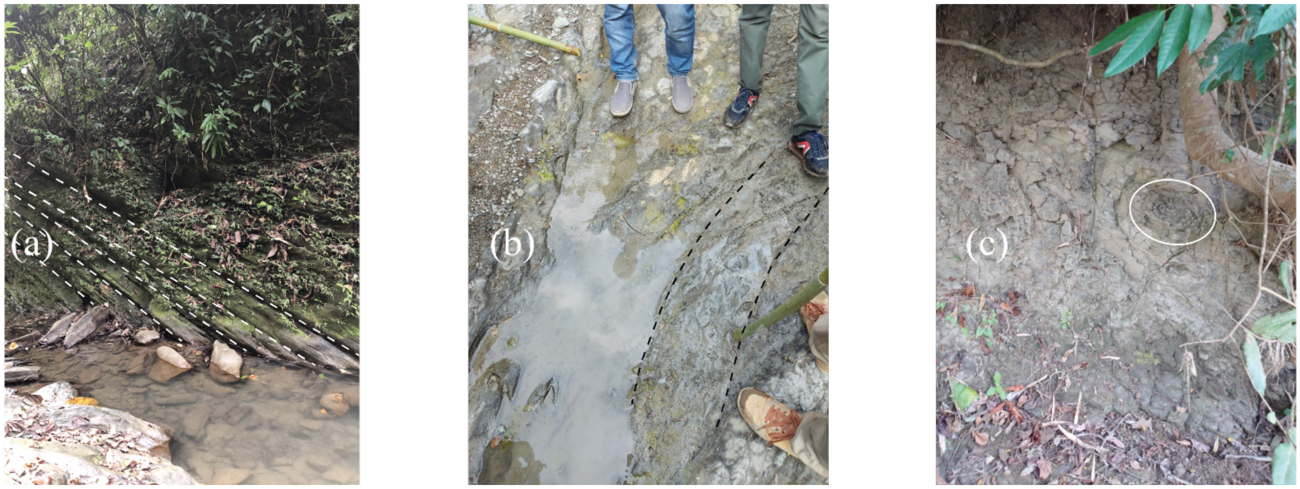

Figure S5: *Stratigraphic column and different attributes in the Risang-Thakurchara section: (a) Repetitive features of sandstone and shale. (Shale is eroded or inter-bedded due to its higher erosional nature); (b) Tectonic rolling and influence of coupling force; (c) Nodular structure in shale;*

c) **Alutila Cave and Army Camp (**23.087778°N, 91.956389°E)

Located in the hilly neighborhood of Khagrachari, Alutila Cave is 100 meters long and has a natural subway-like design with water flowing at the bottom.

Sedimentary features and facies: Coarse-grained sandstone; Heterolithic bed; Massive sandstone; Trough cross-bedding; Parallel laminated bed; Load cast; Climbing ripples; Flame structure; Erosional base; Micro cross lamination; Convolution; Vertical joints; Sand injectites; Vertical stacked channels.

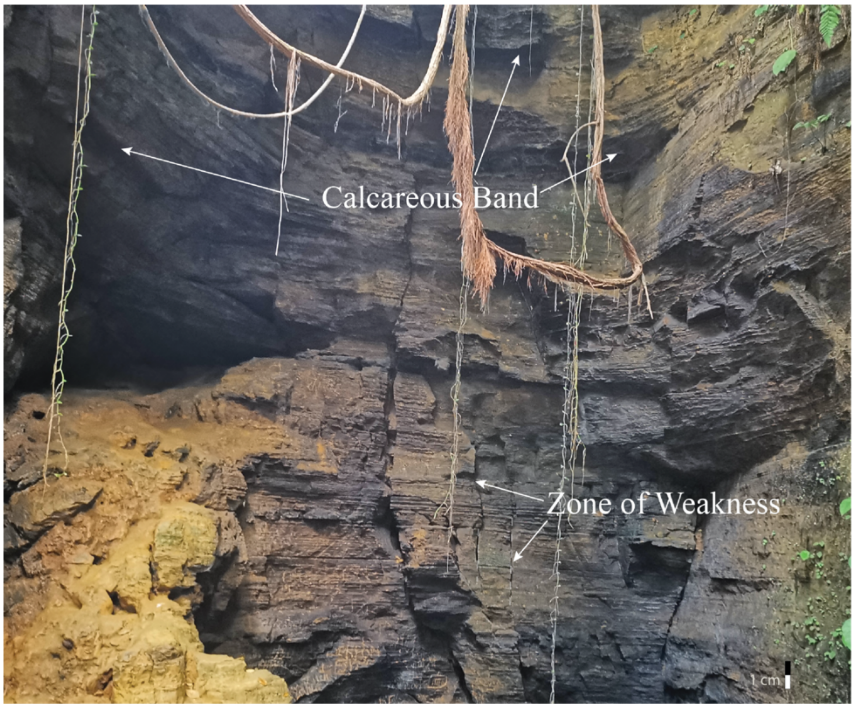

Figure S6: *Wall section of Alutila cave displaying the zone of weakness and calcareous band.*

d) Dhoilachara-Bangmara (23.062778°N, 91.941389°E - 23.056111°N, 91.914722°E)

The highest and lowest elevations along the traverse path were 571 feet and 180 feet, respectively, according to KML image analysis. The overall distance of this segment was more than 12 km and most of the traversing path was acquainted along the stream.

Sedimentary features and facies: Claystone, siltstone, mudstone, shale, and thinly laminated shale; Massive, calcareous, and laminated sandstones; Nodular, pro-deltaic, and fissile shales; Conglomerate; Drag fold and crisscross joints; Yellowish brown massive sand unit; Gas sand.

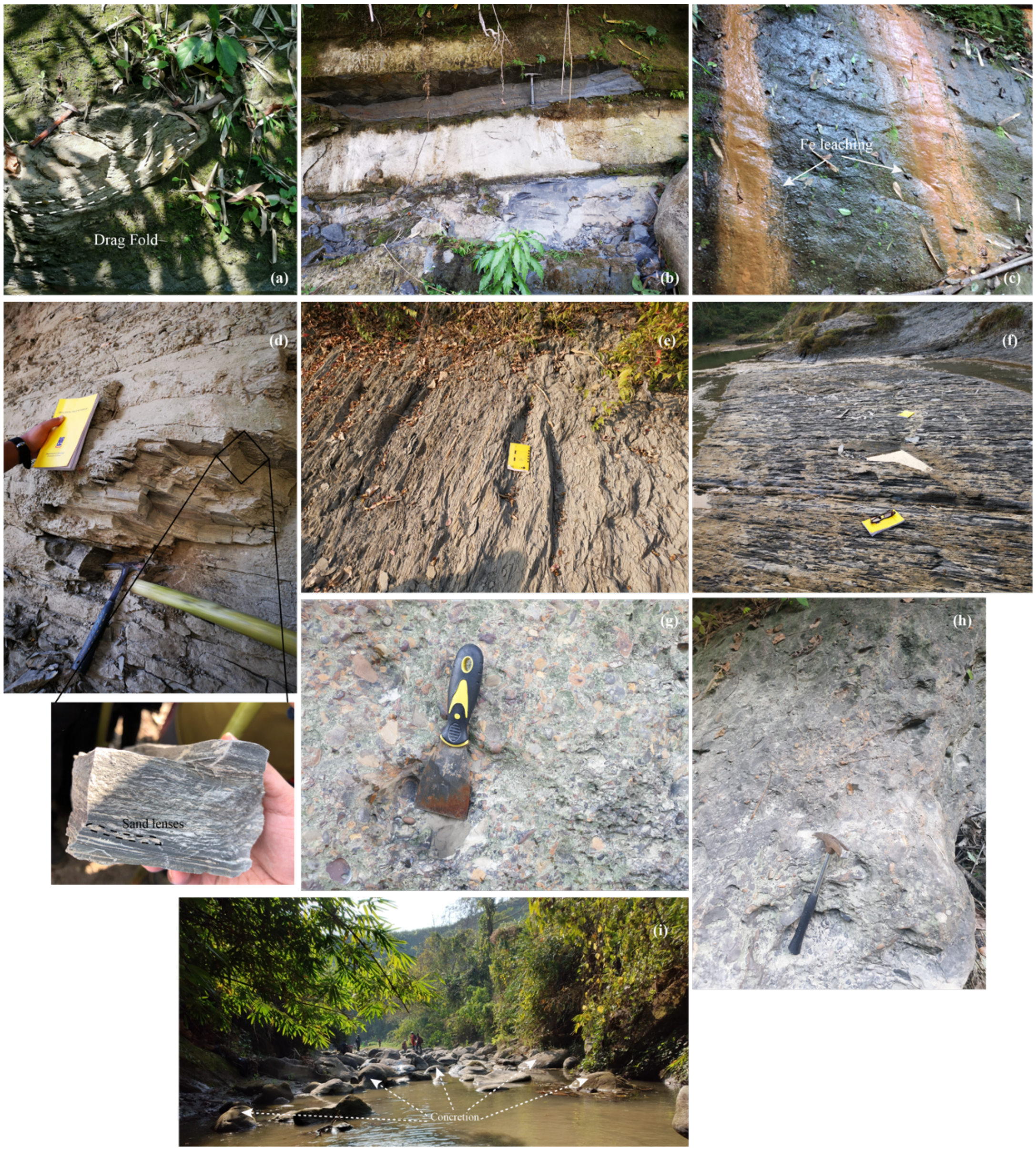

Figure S7: *Different notable features and discrete lithocoloumn of Dhoilachara-Bangmara Section: (a) Small scale drag fold indicating active tectonism; (b) Claystone/mudstone; (c) Fe leaching in the exposed rocks; (d) Laminated shale with sand lenses; (e) Paper thin laminated shale; (f) UMS or UMS eq. shale (Reassessed is recommended as confused with thinly laminate silty-shale); (g)+(h) Lag deposits; (i) Presence of concretion.*

e) **Matiranga-Alutila Road Cut** (23.079167°N, 91.939444°E - 23.080833°N, 91.950278°E)
The total traversing distance of this section was ~4km and KML image analysis showed a relatively higher elevation from MSL. The highest and lowest elevation was 885 feet and 644 feet respectively.

Sedimentary features and facies: Heterolithic beds; Trough cross-bedding; Ripple laminated beds; Parallel laminated beds; Channel sand; Stacked channel bodies; Lag deposits; Injectites; Micro cross lamination; Flame structure; Convolution structure; Channel fill clay; Soft deformed sediment; Laterally extensive channel sand.

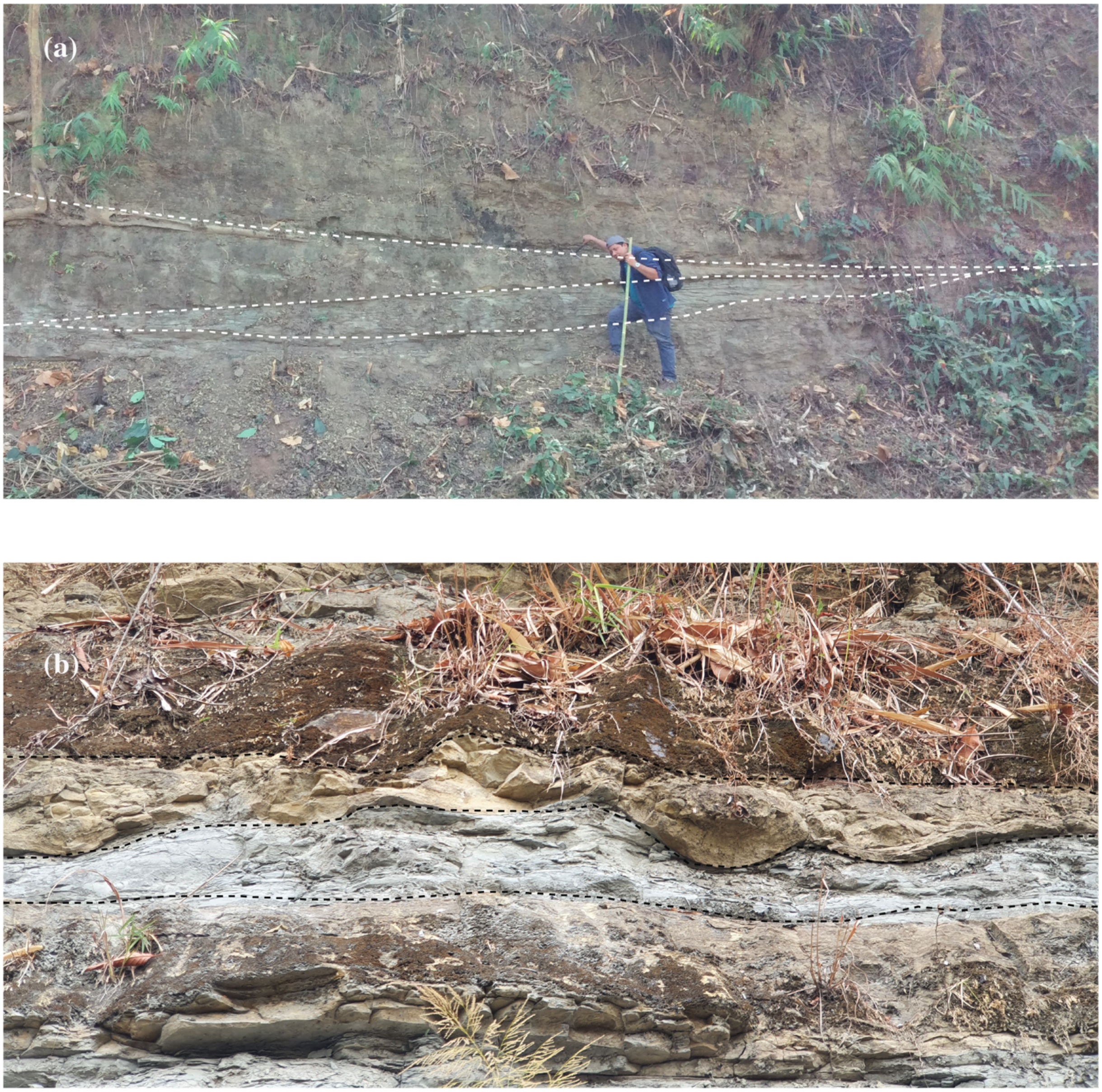

Figure S8: *(a) Amalgamated channel sand (pinching out) in the Matiranga-Alutila road cut section. (b) Channel lag deposits in the Matiranga-Khagrachari road cut section*

S2 Bed Orientation

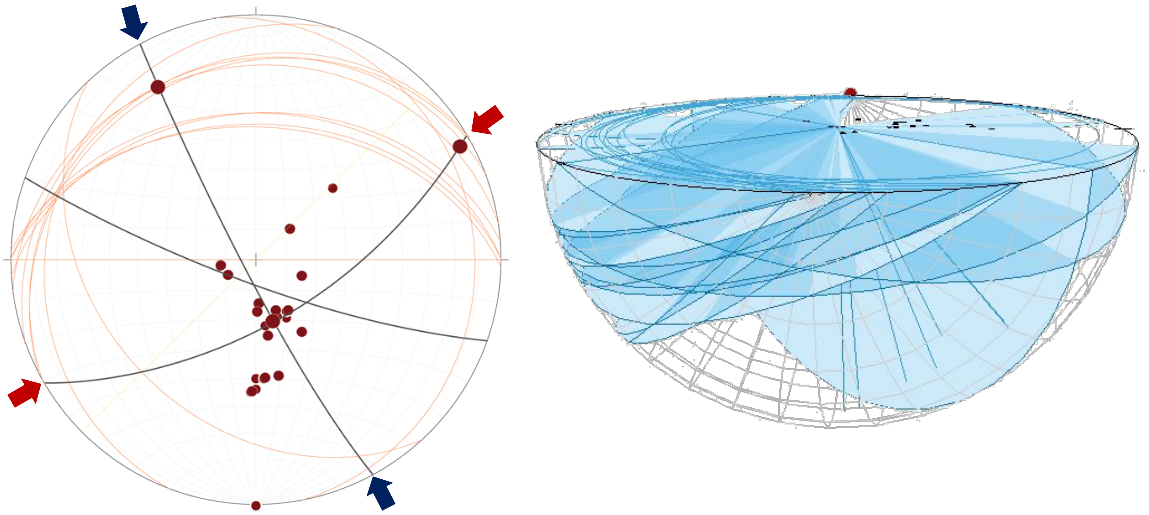

Figure S9: *(a) Stereonet projection and (b) 3D orientation of the bedding plane of Changotaung Anticline with the direction of compressional forces. The figures made with visible geology map (https://app.visiblegeology.com/)*
