# Supplementary material for: High-resolution lithostratigraphy and reconnaissance sedimentology of Changotaung structure, Chittagong Tripura fold belt, Bengal Basin, Bangladesh
Source: Sci Rep. 2023 Oct 18;13:17727. doi: 10.1038/s41598-023-43810-7 (PMC10584892; doi:10.1038/s41598-023-43810-7)
Supplement: Supplementary file 4 — Supplementary Information 4. [file 41598_2023_43810_MOESM4_ESM.pdf]

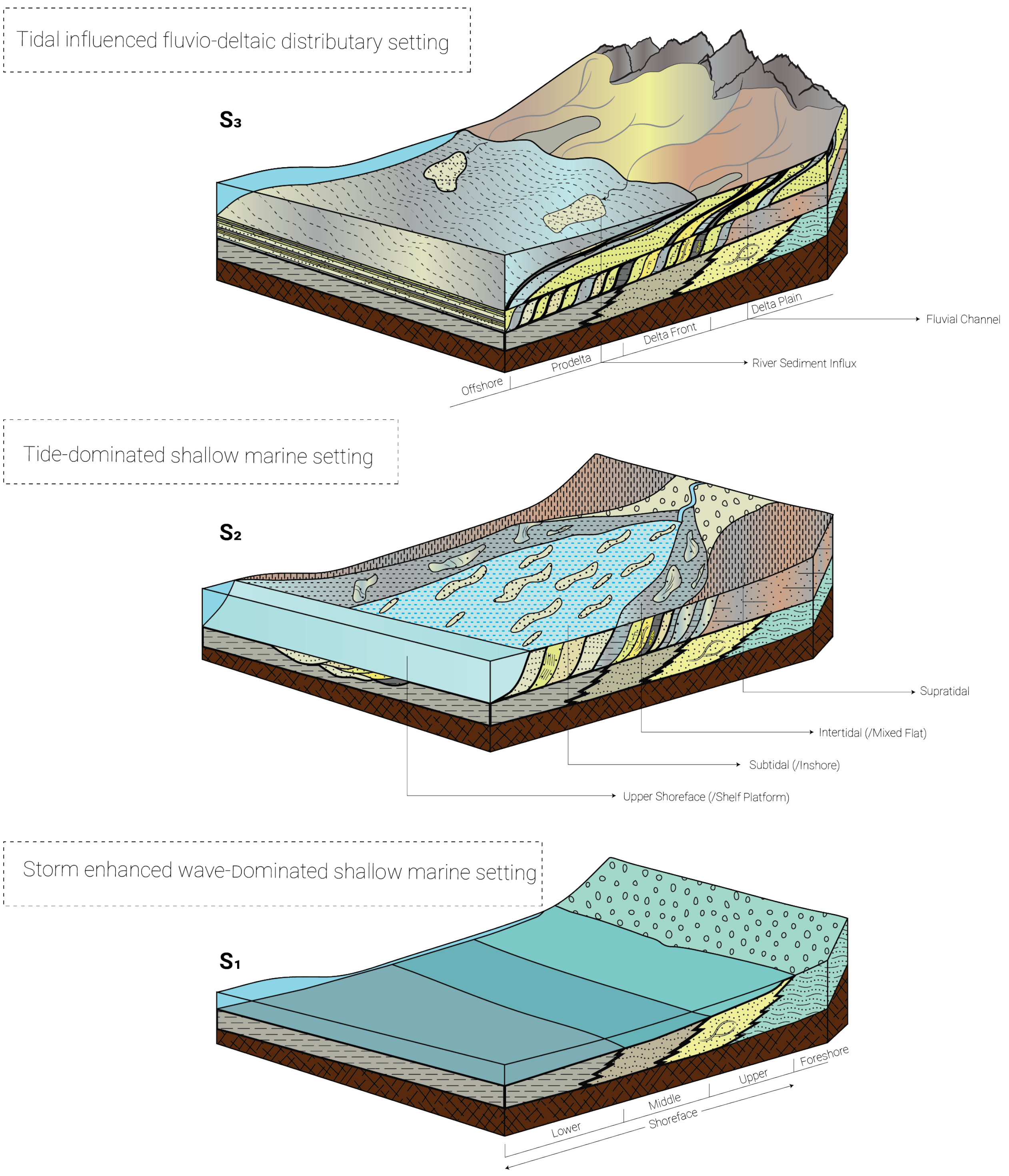

A Schematic three-stage proposed depositional settings for the Upper Surma Group at CTFB region in Bengal Basin. S1 (Older) to S3 (Younger) represent the environment from wave-dominated shallow marine settings to fluvio-deltaic distributary settings with tidal influences at each stage.
